# Repurposing First-Row Transition Metal Carbon Dioxide Reduction Electrocatalysts for Electrochemical Carboxylation of Benzyl Chloride

**DOI:** 10.1021/acsorginorgau.4c00051

**Published:** 2024-10-20

**Authors:** Pornwimon Kongkiatkrai, Thana Anusanti, Teera Chantarojsiri

**Affiliations:** Department of Chemistry and Center of Excellence for Innovation in Chemistry, Faculty of Science, Mahidol University, Bangkok, 10400, Thailand

**Keywords:** CO_2_ Utilization, Electrocarboxylation, CO_2_−Cross Coupling, Electrocatalysis, Electrosynthesis, CO_2_ Reduction

## Abstract

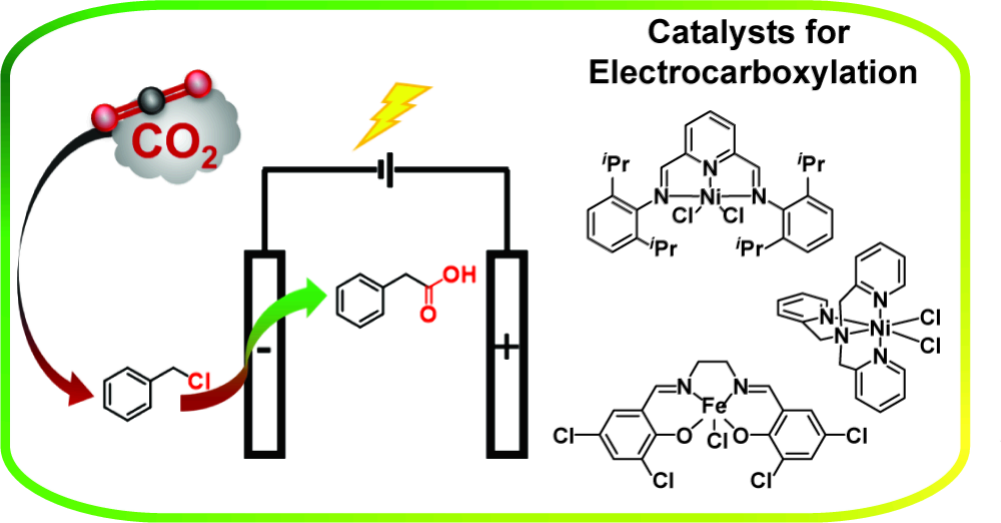

Carbon dioxide (CO_2_) is an abundant and useful C_1_ feedstock for electrocarboxylation, a process that incorporates a carboxyl moiety into an organic molecule. In this work, three first-row transition metal CO_2_ reduction electrocatalysts, NiPDI^iPr^ (**1**), NiTPA (**2**), and Fe(salenCl_4_) (**3**), were explored as electrocarboxylation catalysts with benzyl chloride as a substrate. The cyclic voltammograms of all three catalysts showed current enhancements in the presence of benzyl chloride under a CO_2_ atmosphere. Introduction of DMAP as additives showed further current enhancement. Electrolyses with one-compartment cell generated a moderate yield of phenylacetic acid. Addition of MgBr_2_ was proven to be crucial to the formation of the carboxylate product. While the yield of carboxylation was moderate, this work showed an example of electrocarboxylation of benzyl chloride without using a metal electrode or sacrificial anode, which could lead to a more sustainable carboxylation methodology.

## Introduction

The escalating levels of atmospheric carbon dioxide (CO_2_) pose a significant environmental challenge, principally contributing to the global climate crisis. While CO_2_ is the most abundant greenhouse gas emitted by human activities and therefore the primary culprit, it also presents a potentially valuable opportunity. CO_2_ can be leveraged as a readily available C_1_ feedstock for chemical synthesis.^1^

CO_2_ has been investigated as a starting material for various chemical transformations. However, its inherent chemical inertness renders it difficult to activate and utilize. This stability originates from its linear molecular structure and the strong double bonds between the carbon and oxygen atoms. These robust bonds make CO_2_ a low-energy, low-reactivity molecule, necessitating significant energy input to break them and reform them into other compounds.^2,3^

In nature, the enzyme Rubisco efficiently fixes CO_2_ from the atmosphere to generate a carboxylate product that enters the Calvin–Benson–Bassham cycle.^4,5^ In organic syntheses, activating CO_2_ for the generation of carboxylate products typically requires high energy input and a water-sensitive Grignard reagent, an organomagnesium compound.^6−8^ Catalysts can be employed to activate CO_2_ and facilitate various transformations, including carboxylation,^9−13^ carbonylation,^12^ and direct reductions to other C_1_ products, such as carbon monoxide (CO), formate, and methanol.^1^

Several review papers have emphasized the potential of CO_2_ as a C_1_ feedstock.^14−16^ Direct CO_2_ reduction can generate multiple products, with CO being the most common product from the two-electron reduction process. While CO is a useful feedstock for the industry, its π-accepting nature can cause product inhibition, especially in the presence of metal complex catalysts.^17,18^ Carboxylation and carbonylation reactions, on the other hand, have received significant interest, leading to the development of several processes aimed at maximizing efficiency and selectivity. Chemical reduction approaches, utilizing organohalides as starting materials, have been traditionally conducted with various metal-based reductants. However, these methods can generate significant waste from reductant byproducts, hindering the process’s sustainability.^19−21^ A more promising alternative is electrocarboxylation, a greener method for carboxylate synthesis from organohalides.

The inherent inertness of CO_2_ often requires the generation of high-energy CO_2_^•–^ species through electrochemical reduction. This radical anion then reacts with the substrate’s C–X bond, forming another high-energy product: a carboxylate species that can deposit on the electrode. This led to the implementation of sacrificial anodes, such as Mg, Al, or Zn metals, believed to aid in stabilizing the carboxylate species and sustaining the catalysis.^22,23^

Using this knowledge in hand, a direct CO_2_ reduction catalyst can also generate CO_2_^•–^ readily available for coupling with benzyl halides as substrates. Benzyl halides can also form benzyl radicals, enabling coupling with CO_2_^•–^. Previous work explored the carboxylation of aryl chlorides using a Ni complex catalyst in the presence of a carbon-based electrode to minimize the use of precious metal electrodes.^24^ This approach demonstrates the potential for replacing sacrificial anodes with the addition of Mg salts for intermediate stabilization.

Nickel complexes supported by the pyridinediimine-type backbone were examined as electrocatalysts for CO_2_ reduction.^25^ Various derivatives have shown promising catalytic current enhancement. Ligands with iPr derivatives showed high current enhancement at the third reduction under a CO_2_ atmosphere, but controlled potential electrolysis exhibited high selectivity toward H_2_ production.^26^ Fe complexes with the pyridinediimine with a hemilabile phosphine ligand showed sequential CO_2_ deoxygenation reactivity via a chemical reductant.^27^ Ni complexes supported by tris(pyridyl)methylamine have been shown to bind with CO_2_. Direct reaction with a bis-μ-hydroxide dinuclear complex generated bridging carbonate species.^28^ Mononuclear Ni with TPA ligand also exhibited electrocatalytic CO_2_ reduction activity, with CO and CO_3_^2–^ as products, suggesting a disproportionation process.^29^ Fe complexes with salen-type ligands were also shown to be active toward CO_2_ electroreduction, with CO as a primary product.^30^

**Chart 1:**
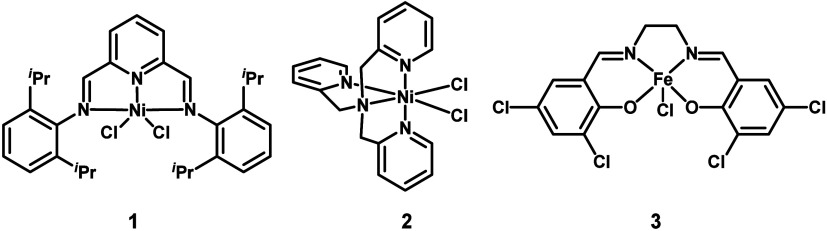
Structure of Electrocatalysts **1**, **2**, and **3** Used for Electrocarboxylation in This Work

Herein, we explore the ability of three first-row transition metal complexes, NiPDI^iPr^ (**1**), NiTPA (**2**), and Fe(salenCl_4_) (**3**), Chart 1, which have been studied as direct CO_2_ reduction catalyst to CO, as catalysts for electrocarboxylation of benzyl chloride in organic media, using a carbon cloth electrode and in the absence of the sacrificial anode and metal electrode.

## Results and Discussion

Complexes **1**, **2**, and **3** were synthesized according to literature procedures, starting with ligand synthesis and metalation with NiCl_2_·6H_2_O and anhydrous FeCl_3_, respectively. Details on the syntheses can be found in the Supporting Information. After recrystallization, all metal complexes **1**, **2**, and **3** were characterized by mass spectrometry, UV–vis, and IR spectroscopy (Figures S1–S13)

**Figure 1:**
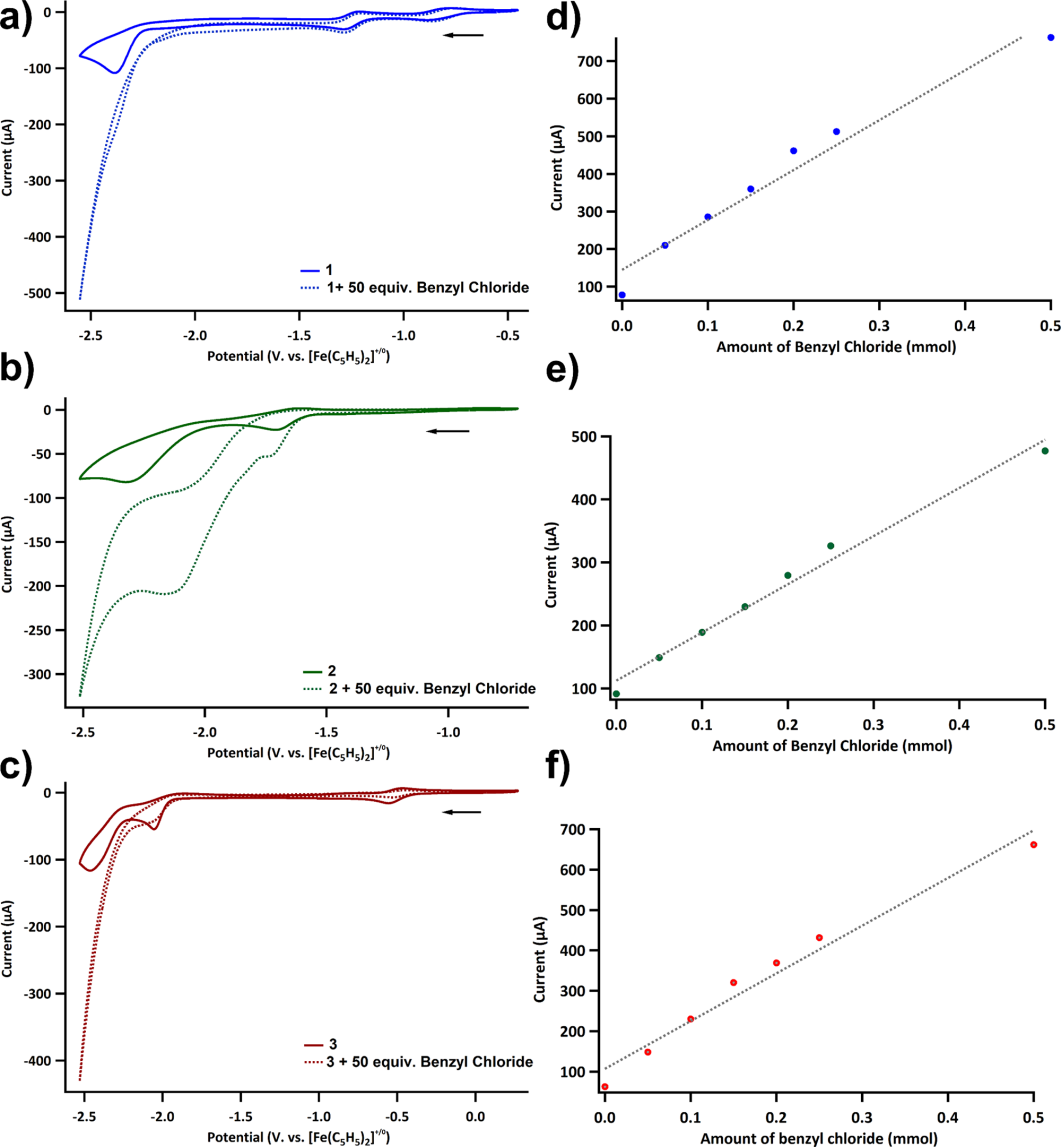
Cyclic Voltammogram under CO_2_ without (solid line) and with (dotted line) benzyl chloride of **1** (a), **2** (b), and **3** (c). Working electrode, counter electrode, and reference electrode are glassy carbon, Pt wire, and Ag/Ag^+^ pseudo-reference electrode, respectively. Cyclic voltammograms were recorded in MeCN with 0.1 M TBAPF_6_ and 1 mM 1, 2, or 3, at a scan rate of 100 mV/s. Peak current and benzyl chloride amount from cyclic voltammetry of **1** (d), **2** (e), and **3** (f) under CO_2_ with varying amounts of benzyl chloride.

In order to screen for electrochemical CO_2_ reduction by cyclic voltammetry, complexes **1**, **2**, and **3** were subjected to cyclic voltammetry and all exhibited catalytic current enhancement under a CO_2_ atmosphere (Figure S14). Addition of 50 equiv of benzyl chloride as a substrate showed current enhancement at the third reduction for complex **1** (Figures 1a, S15), supposedly ligand-based reduction, the second reduction, suggesting Ni(I) active species for complex **2** (Figures 1b, S16), and the second reduction, Fe(I) species, for complex **3** (Figures 1c, S17). All three complexes showed a linear dependence of current on the amount of substrate, suggesting first-order kinetics with benzyl chloride (Figure 1d–f).

Benzyl halide substrates often undergo electrocarboxylation with the aid of a sacrificial anode.^14^ However, the stoichiometric amount of metal electrode consumed during this process could render it unsustainable. Previous reports have established that using Mg salts as additives helps stabilize the intermediate, similar to a sacrificial anode. In this work, MgBr_2_ was employed as a Lewis acid additive to enhance electrocarboxylation. 6-dimethylaminopyridine (DMAP) has been found to be beneficial for various Ni-catalyzed carboxylations. As a result, the effects of DMAP on the electrocatalysis were further explored using cyclic voltammetry.

**Figure 2:**
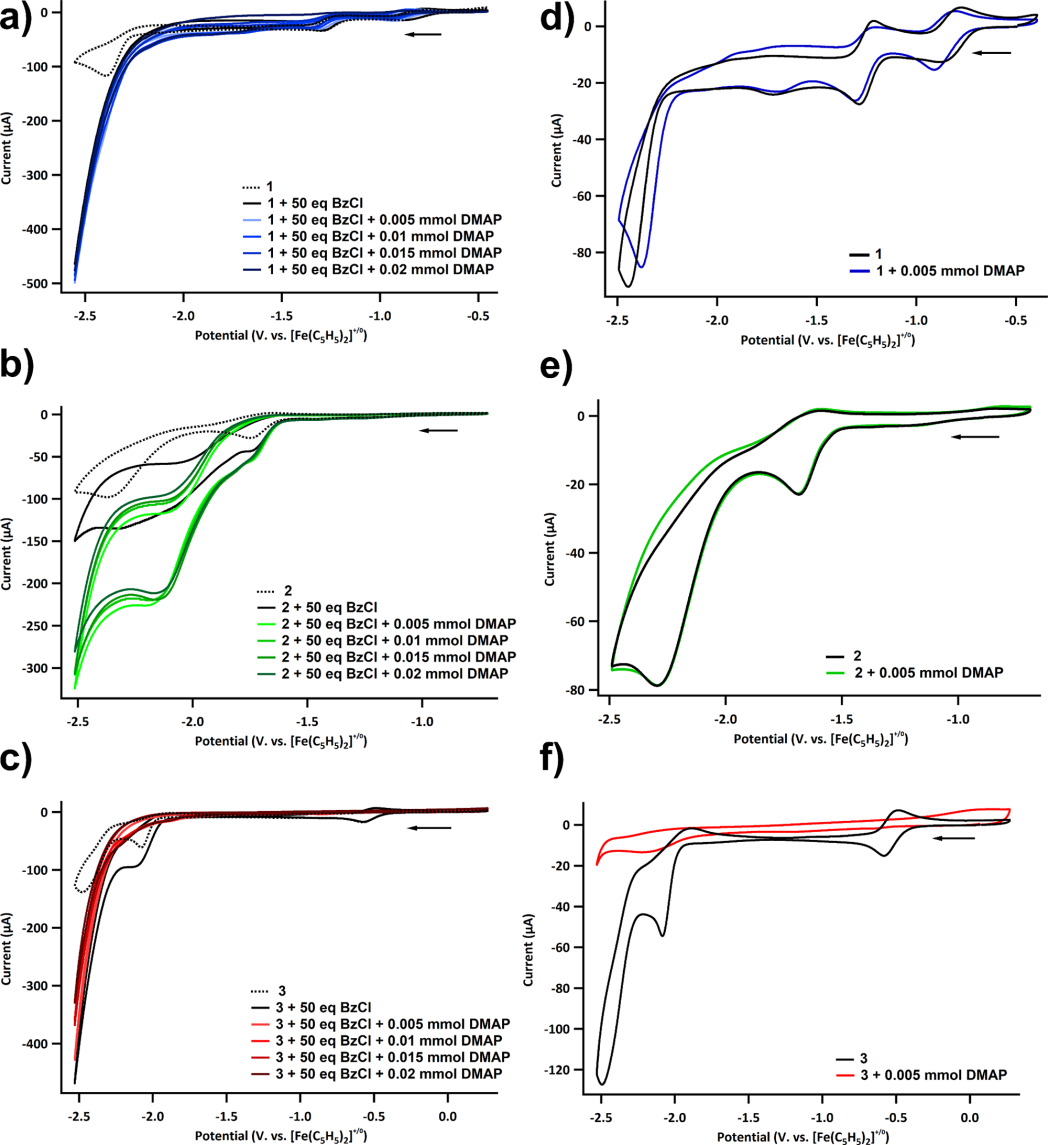
Cyclic voltammogram under CO_2_ with (dotted line) and without (solid line) benzyl chloride of **1** (a), **2** (b), and **3** (c) with additional DMAP. Cyclic voltammogram under CO_2_ with and without DMAP of **1** (d), **2** (e), and **3** (f). Working electrode, counter electrode, and reference electrode are glassy carbon, Pt wire, and Ag/Ag^+^ pseudo reference electrode, respectively. Cyclic voltammograms were recorded in MeCN with 0.1 M TBAPF_6_ and 1 mM of **1**, **2**, or **3**, at the scan rate of 100 mV/s.

Addition of DMAP to complexes **1**, **2**, and **3**, in the presence of CO_2_ and benzyl chloride, showed varying amounts of current enhancement. Complex **1** with 1 to 4 equiv of DMAP showed a 4-fold current enhancement (Figure 2a), while complex **2** showed approximately a 2-fold increase with 1 to 4 equiv of DMAP (Figure 2b). Complex **3**, on the other hand, showed a reduced current in the presence of DMAP (Figure 2c). Exploration of complex **3** with DMAP under CO_2_, without substrate, revealed the loss of the Fe(III/II) redox couple (Figure 2f), suggesting that the metal complex might not be stable. With these results in hand, electrolyses of benzyl chloride and CO_2_, catalyzed by complexes **1**, **2**, and **3**, were performed.

Electrolyses of benzyl chloride under a CO_2_ atmosphere were conducted first in acetonitrile, with **1**, **2**, or **3** as catalysts. The one-compartment cell equipped with two carbon cloth electrodes and a flow of 1 atm of CO_2_ was employed (Figure S18). A constant current of 5 mA was delivered by a power supply, and the current was held constant for 4 h. Unfortunately, only a small amount of phenylacetic acid, the carboxylation product, was produced. We speculated that the low yield might be caused by the low solubility of the Mg salts (Mg(OTf)_2_, MgBr_2_, and MgCl_2_) used as additives, as previous reports have shown Mg salts to be crucial in electrocarboxylation. As a result, we turned to dimethylformamide (DMF) and *N*-methylpyrrolidone (NMP), which are also commonly used in electrocarboxylation or electrocatalysis.^31^ Soluble products observed in this process are 1) phenyl acetic acid, the carboxylation product, 2) bibenzyl from self-coupling of benzyl radical, and 3) benzyl alcohol from the hydrolysis of benzyl radical. It is important to note that the gaseous products from all electrolyses were not identified due to the lack of instrumentation for gas analysis. Comprehensive examinations of all products and mechanistic investigations will be conducted in subsequent studies.

Table 1 summarizes the results from the electrolyses. Complex **1** in NMP, with DMAP and MgBr_2_ showed production of 55% yield of phenylacetic acid with 79% selectivity (entry 1, Table 1). Small amounts of benzyl alcohol (4%) and bibenzyl (3%) were generated. Only 37% of the total charges went toward the generation of phenylacetic acid. The remaining charges were likely spent producing gaseous products from CO_2_, proton reduction, or decomposition of the benzyl chloride. Increasing the catalyst loading surprisingly resulted in a decrease in the percent yield and selectivity of phenylacetic acid (entries 2–3, Table 1). DMAP was proven crucial for complex **1** as a catalyst, as its absence dropped the yield to 9% with lower conversion as well (entry 4, Table 1). A control experiment without DMAP or complex 1 showed only 5% phenylacetic acid with only 24% conversion of substrate, establishing the background reaction (entry 5, Table 1). Performing the experiment under argon (Ar) showed no production of phenylacetic acid and only trace amounts of benzyl alcohol, presumably from radical formation followed by hydrolysis during workup (entry 6, Table 1). Changing the supporting electrolyte from tetrabutylammonium hexafluorophosphate (TBAPF_6_) to tetrabutylammonium bromide did not yield any improvement (entry 7, Table 1). Changing the solvent to DMF also decreased the yield while maintaining high conversion (65%), suggesting decomposition of the substrates (entry 8, Table 1). Mixing DMF and NMP yielded similar results (entry 9, Table 1). MgBr_2_ was proven important, as reactions without MgBr_2_ showed lower amounts of the carboxylation product (entry 10, Table 1), and switching from MgBr_2_ to MgCl_2_ also showed lower yields of phenylacetic acid (entry 11, Table 1).

**Table 1:**
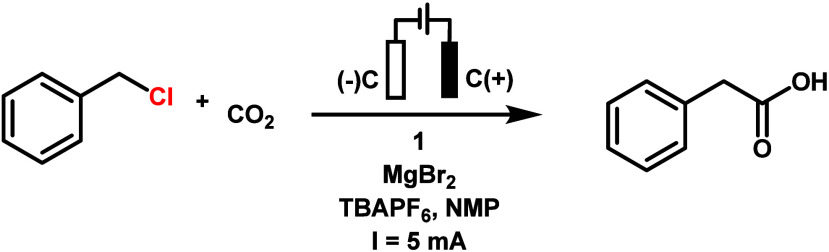
Conditions of Electrocarboxylation of Benzyl Chloride by **1**

		Average% Yield^a^						
Entry	Deviation from Standard Condition	Phenyl acetic acid	Bibenzyl	Benzyl alcohol	Conversion (%)	Selectivity for phenyl acetic acid (%)	FE COOH (%)	TON
1	2 mol % of **1** and 2 mol % of DMAP	55	3	4	70	79	37.1	27.7
2	5 mol % of **1** and 5 mol % of DMAP	17	2	4	62	27	11.2	8.3
3	10 mol % of **1** and 10 mol % of DMAP	15	3	5	69	22	10.2	7.6
4	2 mol % of **1** without DMAP	9	trace	9	45	20	6.1	4.5
5	without **1** and DMAP	5	trace	8	24	21	3.4	2.5
6	2 mol % of **1** and 2 mol % of DMAP under Ar	0	trace	7	35	0	0	0
7	2 mol % of **1** and 2 mol % of DMAP TBABr	34	trace	6	64	54	22.9	17.1
8	2 mol % of **1** and 2 mol % of DMAP in DMF	6	trace	11	65	10	4.3	3.2
9	2 mol % of **1** and 2 mol % of DMAP in DMF/NMP (1:1)	13	trace	7	54	23	8.4	6.3
10	2 mol % of **1** and 2 mol % of DMAP without MgBr_2_	22	1	6	50	43	14.4	10.7
11	2 mol % of **1** and 2 mol % of DMAP with MgCl_2_	32	1	5	55	58	21.5	16.1

a Yield reported was an average of three or more runs.

Complex **2** behaved differently from complex **1** and showed less response to DMAP additives (entry 4, Table 2). Increasing the catalyst loading did not increase the yield of the product (entries 1–3, Table 2). Similar to complex **1**, complex **2** did not perform better with tetrabutylamminoum bromide (TBABr) as a supporting electrolyte (entry 5, Table 2) or without the addition of MgBr_2_ (entry 8, Table 2). DMF and 50% NMP in DMF also rendered the catalysis less effective (entries 6–7, Table 2). A control experiment without CO_2_ also yielded no carboxylic acid product (entry 10, Table 2). Complex **3** showed an almost linear increase in product yield with increased catalyst loading (entry 1–3, Table 3). However, taking the best condition (10 mol % catalyst loading) to optimize further yielded similar results to complexes **1** and **2**: the presence of MgBr_2_ is necessary for higher yield and selectivity (entry 7, Table 3). Electrolysis under Ar showed no phenylacetic acid product, proving the role of CO_2_ in the reaction (entry 9, Table 3). Complex **1** exhibited superior electrocarboxylation reactivity compared to complexes **2** and **3**, respectively. This enhanced activity might be attributed to the ability of all three catalysts to perform direct electroreduction of CO_2_ to CO, as **3** showed a higher reactivity to **2** and **1**, respectively.

**Table 2:**
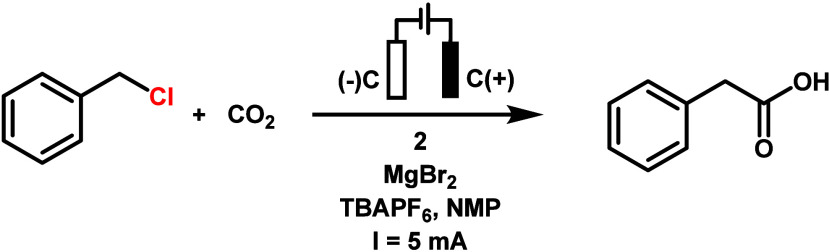
Conditions of Electrocarboxylation of Benzyl Chloride by **2**

		Average% Yield^a^						
Entry	Deviation from Standard Condition	Phenyl acetic acid	Bibenzyl	Benzyl alcohol	Conversion (%)	Selectivity for phenyl acetic acid (%)	FE COOH (%)	TON
1	2 mol % of **2**	26	0	9	57	46	17.6	13.1
2	5 mol % of **2**	10	0	5	35	27	6.4	4.7
3	10 mol % of **2**	14	1	5	53	27	9.6	7.2
4	2 mol % of **2** with 2 mol % of DMAP	25	1	5	70	35	16.8	12.5
5	2 mol % of **2** with TBABr	15	0	4	46	32	9.8	7.3
6	2 mol % of **2** in DMF	7	0	5	76	9	4.7	3.5
7	2 mol % of **2** in DMF/NMP (1:1)	13	0	5	49	28	9.0	6.7
8	2 mol % of **2** without MgBr_2_	9	trace	5	48	19	6.2	4.6
9	2 mol % of **2** with MgCl_2_	22	trace	5	63	35	14.7	10.9
10	2 mol % of **2** in Ar	0	1	7	40	0	0	0

a Yield reported was an average of three or more runs.

**Table 3:**
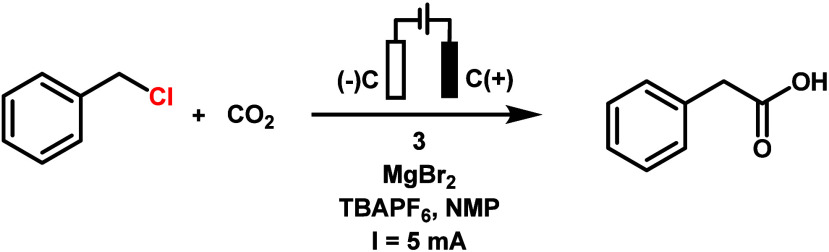
Conditions of Electrocarboxylation of Benzyl Chloride by **3**

		Average% Yield^a^						
Entry	Deviation from Standard Condition	Phenyl acetic acid	Bibenzyl	Benzyl alcohol	Conversion (%)	Selectivity for phenyl acetic acid (%)	FE COOH (%)	TON
1	2 mol % of **3**	8	0	7	44	17	5.1	3.8
2	5 mol % of **3**	14	0	4	44	32	9.6	7.1
3	10 mol % of **3**	30	0	5	53	56	19.9	14.9
4	10 mol % of **3** with TBABr	20	0	4	46	43	13.1	9.8
5	10 mol % of **3** in DMF	5	0	5	45	12	3.6	2.7
6	10 mol % of **3** in DMF/NMP (1:1)	10	0	5	48	21	6.9	5.2
7	10 mol % of **3** without MgBr_2_	15	0	5	42	37	10.4	7.7
8	10 mol % of **3** with MgCl_2_	17	0	5	50	35	11.5	8.6
9	10 mol % of **3** in Ar	0	0	5	28	0	0	0

a Yield reported was an average of three or more runs.

To gain clues about the reaction mechanism, electrolyses catalyzed by complexes **1**, **2**, and **3** were performed with the addition of butylated hydroxytolulene (BHT), the radical inhibitor, under the highest yield conditions. All three catalysts showed no carboxylation product. Instead, a small amount of benzyl alcohol was solely produced. This result suggests the possible presence of a benzyl radical intermediate that was hydrolyzed during the acidic workup to yield an alcohol product. Reactivity studies of **1**, **2**, and **3** with benzyl chloride by cyclic voltammetry suggested the possibility of dehalogenation of benzyl chloride, as shown by the current enhancement of metal complexes with benzyl chloride under Ar (Figures S19–S21). Previous works also demonstrated dehalogenation as the first step for electrocarboxylation of organohalides.^32,33^ However, the attempt to trap the intermediate with TEMPO only resulted in a small amount of benzyl alcohol as a product.

From the results of electrocatalysis by all three complexes, the presence of MgBr_2_ salts was crucial to the carboxylation. Previous works suggested that utilization of Mg^2+^ to stabilize the carboxylate intermediate, instead of using a sacrificial Mg anode, can also promote the reaction. The redox couple of Br_2_ to Br^–^ can also be important as an anodic reaction as MgCl_2_ gave less product yield. It is also important that the Mg salt is soluble in the solvent during catalysis. While cyclic voltammetry showed high catalytic current in MeCN, MgCl_2_ or MgBr_2_ are barely soluble in MeCN, resulting in no carboxylation product. NMP, the solvent with the highest solubility of Mg halide salts, yielded the most product in all three catalysts. It is also worth mentioning that only Br_2_ to Br^–^ alone is not sufficient as TBABr as the electrolyte failed to enhance the production of phenylacetic acid. Therefore, the presence of Mg^2+^ is crucial to stabilize the reactive carboxylate.

The addition of DMAP enhanced the carboxylation reaction only when **1** was used as a catalyst. In previous works with other Ni-catalyzed carboxylation, DMAP was added as an extra ligand, together with di-*tert*-butylpyridine to increase electron donor to the metal center, enabling higher basicity of reduced species.^24^ In this case, it is highly probable that **1**, as a tridentate ligand, is more susceptible to forming a complex with additional DMAP while still maintaining enough opening site to coordinate with the starting material. While **2** and **3** are Ni and Fe complexes with tetradentate ligand which might not provide open coordination sites for the carboxylation after the addition of DMAP. Furthermore, the cyclic voltammetry also showed that **1** possesses 3 consecutive reductions, with the two metal-based and the third ligand-based reduction, which could provide more reducing power to the substrate to generate benzyl radical necessary for the carboxylation.

It is worth noting that even though the yield of phenylacetic acid in this work is not particularly high, the electrocarboxylation of benzyl chloride is quite challenging. To the best of our knowledge, most successful electrocarboxylation of benzyl chloride utilized metal electrodes, often precious metals, and sacrificial electrodes.^9,14^ Utilizing abundant first-row transition metal catalysts can aid in the production of phenylacetic acid with only carbonaceous electrodes. Further detailed mechanistic studies as well as the gaseous product analysis would be performed in the next step to gain more insights into this electrocatalysis process.

## Conclusions

To conclude, we have demonstrated the viability of repurposing CO_2_ reduction electrocatalysts as electrocarboxylation catalysts when benzyl chloride is present as a substrate. The yield of phenylacetic acid in different catalysts can be improved upon adding additives such as MgBr_2_ and DMAP. The addition of BHT suppressed the reaction, suggesting the involvement of a radical intermediate. In the absence of CO_2_, no carboxylation product was formed, confirming that the source of the carboxyl group is the CO_2_ electrocarboxylation process.

## Data Availability

The data underlying this study are available in the published article and its Supporting Information.

## Supporting Information Available

Information on syntheses and characterizations of compounds, including NMR, UV–visible, and IR spectra, and additional details on materials, methods, and experimental setup (PDF)
