# Supplementary material for: Repurposing First-Row Transition Metal Carbon Dioxide Reduction Electrocatalysts for Electrochemical Carboxylation of Benzyl Chloride
Source: ACS Org Inorg Au. 2024 Oct 20;4(6):620–7. doi: 10.1021/acsorginorgau.4c00051 (PMC11621952; doi:10.1021/acsorginorgau.4c00051)
Supplement: Supplementary file 1 — gg4c00051_si_001.pdf [file gg4c00051_si_001.pdf]

Supporting Information

Repurposing First-row Transition Metal Carbon Dioxide Reduction Electrocatalysts for Electrochemical Carboxylation of Benzyl Chloride

Pornwimon Kongkiatkrai, Thana Anusanti, Teera Chantarojsiri*

Department of Chemistry and Center of Excellence for Innovation in Chemistry, Faculty of Science, Mahidol University, Bangkok, 10400, Thailand

Corresponding Author

*Teera Chantarojsiri, email: teera.cha@mahidol.edu

Table of Contents

General Information S3

1. Syntheses S4
2. Characterizations S9
   NMR Spectroscopy S9
   Mass Spectrometry S15
   UV-vis and IR Spectroscopy S20
3. Electrochemistry Experimental Procedure S21
4. References S30

General Information

All chemicals were purchased from commercial sources and used as received without further purification. All syntheses were performed under ambient atmosphere, unless otherwise noted. Solvents were dried over 3A molecular sieve before use. All NMR spectra were recorded on Bruker Ascend 400 high-resolution magnetic resonance spectrometer (400 MHz). Chemical shifts were reported in ppm (part per million) using residual solvent peaks as a reference ($CDCl_3$ ^1H δ 7.26). Electrospray Ionization Mass spectra (ESI-MS) of compounds were obtained in positive-ion mode using MeCN (HPLC grade) as a solvent in positive-ion mode on Bruker compact QTOF mass spectrometer with Bruker Compass Data Analysis (Version 6.0) system (Bruker Daltonics GmbH & Co.KG). UV-vis spectroscopy experiments were performed by Shimadzu UV-2600 UV-vis spectrophotometer. ATR-FT-IR spectroscopy were collected by Perkin-Elmer Frontier. Electrochemical measurements were performed by CH Instrument model 620E voltammetric analyzer using three-electrode setup: glassy carbon disk electrode (3 mm Diameter) as a working electrode, Ag/Ag^+ electrode (0.1 M [TBA][PF_6] in MeCN) as a pseudoreference electrode and platinum wire as a counter electrode. All electrodes for cyclic voltammetry were purchased from CH Instruments, Inc. (Texas, USA). The electrochemical experiments were done under Ar or CO_2 at room temperature. Ferrocene/ferrocenium couple were used as an external standard. The electrolysis experiments were performed using WANPTEX GPS 305D Power supply as the electrical source. The current was set using a multimeter connected between the electrical source and the electrodes. The one-compartment 2-neck glass cell was equipped with carbon cloth electrodes (carbon cloth 1071 HCB from Fuel Cell Store (Texas, USA), 1×2 cm size) for both working and counter electrodes under Ar or CO_2. The area of submerged carbon cloth electrodes was 1 cm^2. Gas chromatography experiments were performed on GC-FID/TCD (7890B) using Agilent Innowax column (30 m x 0.25 mm x 0.25 μm file thickness) with 1,3,5-trimethoxybenzene as an internal standard.

1. **Syntheses**

Synthesis of Dimethyl-2,6-Pyridine dicarboxylate

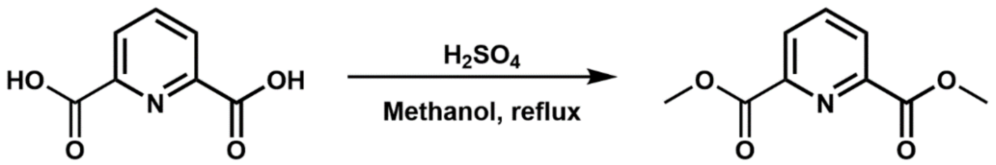

2,6-Pyridine dicarboxylic acid (10 g, 60 mmol, 1 equiv.) was dissolved in methanol (40 mL). Sulfuric acid (2 mL, 360 mmol, 6 equiv.) was added to the mixture and refluxed for 24 hours. The reaction mixture was then cooled to room temperature, leading to crystallization of white crystalline products. In the purification process, the crystalline solid mixture was further concentrated. The crystals were filtered and washed with a small amount of cold MeOH followed by Et_2O. The final product yielded 9.49 g of white solid. (81%).[1]

^1H NMR (400 MHz, $CDCl_3$): δ 8.32 (d, J = 7.8 Hz), 8.03 (dd, J = 8.0, 7.6 Hz), 4.03 (s).

Synthesis of 2,6-Pyridine dimethanol

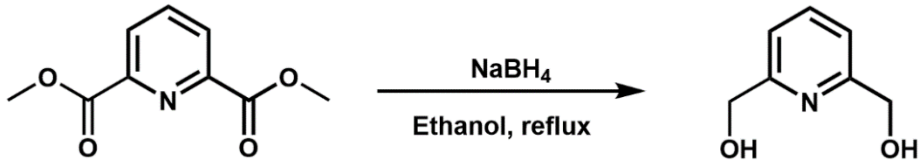

Dimethyl-2,6-Pyridine dicarboxylate (2.0185 g, 10.34 mmol, 1 equiv.) was added to dried EtOH (50 mL). $NaBH_4$ (1.79 g, 47.3 mmol, 4.5 equiv.) was then slowly added to the mixture. The reaction was heated using an oil bath under reflux at 90 °C for 4 hours. EtOH was removed under reduced pressure until completely dried. A saturated solution of K_2CO_3 (60 mL) was added to the white solid. The mixture was stirred at 80 °C for 2 hours. The final product was extracted by DCM. Solvent was removed under reduced pressure. The purification of the product was performed by column chromatography of 10% MeOH in DCM. 2,6-Pyridine dimethanol appeared as white crystalline solid with up to 78.6 % yield. [2]

^1H NMR (400 MHz, $CDCl_3$): δ 7.70 (t, J = 7.7 Hz, 1H), 7.19 (d, J = 7.7 Hz, 2H), 4.78 (s, 4H), 3.38 (s, 2H).

Synthesis of 2,6-Pyridine dicarboxaldehyde

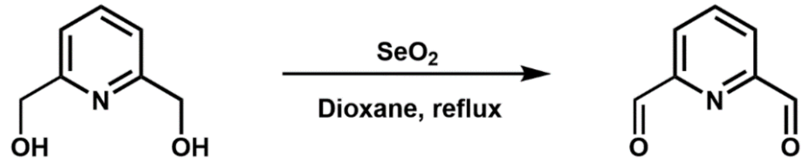

2,6-Pyridine dimethanol (6.6127 g, 47.5 mmol, 1 equiv.) was added to dioxane (50 mL). Then, SeO_2 (5.3552 g, 47.5 mmol, 1 equiv.) was also added into the mixture. The mixture was refluxed for 4 hours. Black solid was removed by filtration while the mixture was still hot. The solvent of the filtrate was removed under reduced pressure to produce white-pinkish crystalline solids. The solid was purified by column chromatography of 20% EtOAc/hexane as eluent. The final product, compound 3, was obtained as white powder weighing 6.0524 g (Yield = 94.3%).[3]

^{1}H NMR (400 MHz, $CDCl_3$): δ 10.17 (s, 2H), 8.19 (d, J = 7.8 Hz, 2H), 8.11 – 8.06 (m, 1H).

Synthesis of 2,6-bis(2,6-diisopropylphenyl imino)methyl pyridine (PDI(^{i}Pr)), 4

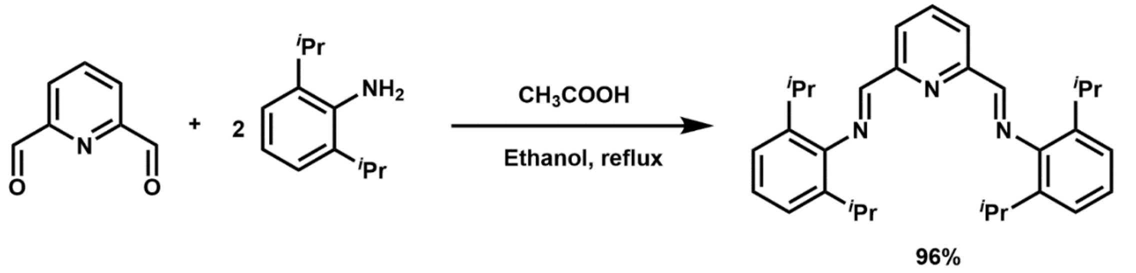

2,6-Pyridine dicarboxaldehyde (0.3337 g, 2.5 mmol, 1 equiv.) was dissolved in ethanol (10 mL). After the addition of 2,6-diisopropylaniline (0.9246 g, 5 mmol, 2 equiv.), 2-3 drops of glacial acetic acid were added dropwise. The reaction was refluxed for 4-5 hours at 85 °C. The mixture was filtered while still hot. The product was obtained as a yellow-green solid (1.0786 g, 96.0%).[4]

^{1}H NMR (400 MHz, $CDCl_3$): δ 8.40 (d, J = 7.8 Hz, 2H), 8.36 (s, 2H), 8.00 (t, J = 7.7 Hz, 1H), 7.21 – 7.10 (m, 6H), 2.98 (sep, J = 8.0 Hz, 4H), 1.19 (d, J = 6.9 Hz, 24H).

$^{13}C\{^{1}H\}$ NMR (101 MHz, $CDCl_3$) δ 162.8, 154.6, 148.4, 137.5, 137.3, 124.7, 123.2, 122.9, 77.5, 77.2, 76.8, 28.1, 23.6.

HRMS (ESI) m/z: $[M + Na]^+$ Calcd for $C_{31}H_{39}N_3Na$ 476.3036; Found 476.3028

HRMS (ESI) m/z: $[M + H]^+$ Calcd for $C_{31}H_{40}N_3$ 454.3217; Found 454.3210

Synthesis of Tris(2-pyridylmethyl)amine (TPA)

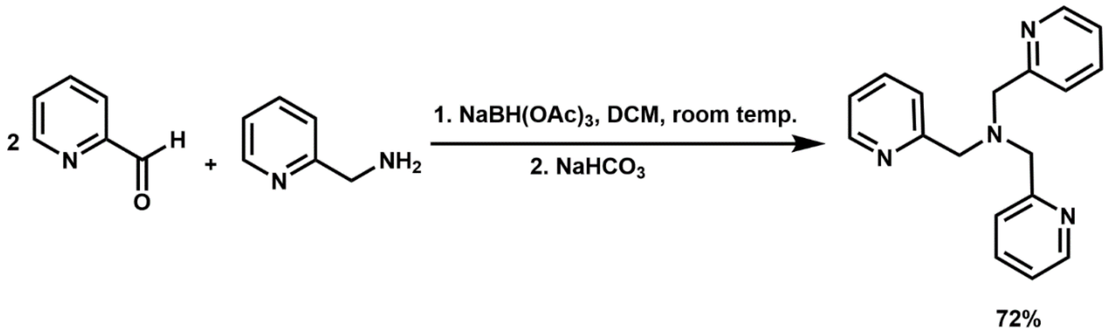

2-Picolylamine (0.4320 g, 4.2 mmol, 1 equiv.) was dissolved in DCM with $NaBH(OAc)_3$ (2.4960 g, 8.4mmol, 2 equiv.). 2-pyridinecarboxaldehyde (0.8988 g, 8.4 mmol, 2 equiv.) was added to the mixture and stirred at room temperature for 18 hours. Saturated solution of $NaHCO_3$ was added into the reaction and stirred for 30 minutes. DCM layer was separated while the aqueous layer was further extracted with EtOAc (3 x 50 ml). The solvent was removed under reduced pressure to yield light yellow oil. Cold Et_2O was added to obtain the solid product. The yellow solid was obtained with 72% yield (0.5200 g). [5]

^{1}H NMR (400 MHz, $CDCl_3$) δ 8.53 (d, J = 4.8 Hz, 3H), 7.65 (td, J = 7.6, 1.8 Hz, 3H), 7.58 (d, J = 7.8 Hz, 3H), 7.14 (ddd, J = 7.1, 4.9, 1.0 Hz, 3H), 3.88 (s, 3H).

$^{13}C\{^1H\}$ NMR (101 MHz, $CDCl_3$) δ 159.5, 149.2, 136.6, 123.1, 122.2, 60.3.

HRMS (ESI) m/z: $[M + Na]^+$ Calcd for $C_{18}H_{18}N_4Na$ 313.1423; Found 313.1423

HRMS (ESI) m/z: $[M + H]^+$ Calcd for $C_{18}H_{19}N_4$ 291.1604; Found 291.1606

Synthesis of N,N'-bis(3,5-dichlorosalicylidene)-1,2-ethylenediamine (SalenCl$_4$)

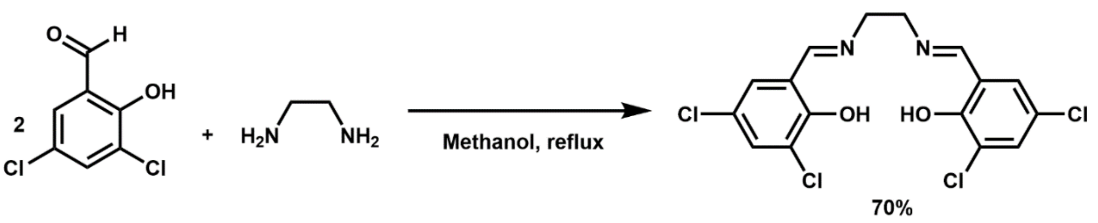

N,N'-bis(3,5-dichlorosalicylidene)-1,2-ethylenediamine was formed by the condensation of 2 equivalent of 3,5-dichlorosalicylaldehyde (0.3888 g, 2 mmol, 2 equiv.) and 1,2-ethylenediamine (0.0601 g, 1 mmol, 1 equiv.) in refluxing methanol for 4 hours. The yellow solid was filtered and washed by cold methanol, giving 70% yield (0.2826 g).[6]

^{1}H NMR (400 MHz, $CDCl_3$) δ 13.95 (s, 2H), 8.28 (s, 2H), 7.40 (d, J = 2.5 Hz, 2H), 7.15 (d, J = 2.5 Hz, 2H), 4.01 (s, 4H).

$^{13}C\{^{1}H\}$ NMR (101 MHz, $CDCl_3$) δ 159.5, 149.2, 136.6, 123.1, 122.2, 60.3.

HRMS (ESI) m/z: $[M + Na]^+$ Calcd for $C_{16}H_{12}Cl_4N_2O_2Na$ 426.9546; Found 426.9548

Synthesis of Ni-PDI(^{i}Pr), 1

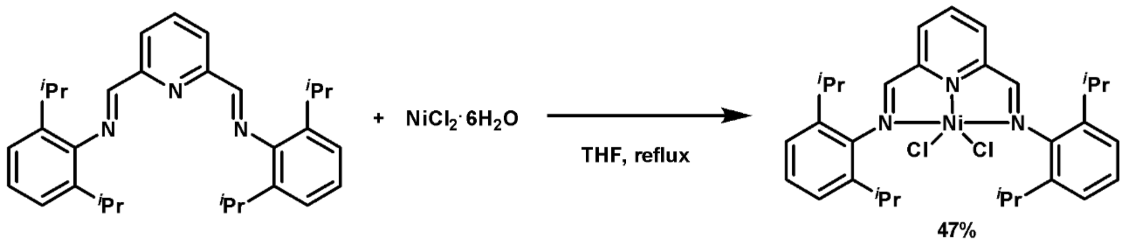

2,6-bis(2,6-diisopropylphenyl imino)methyl pyridine (0.5005 g, 1.10 mmol, 1 equiv.) was dissolved in THF. $NiCl_2.6H_2O$ (0.2654 g, 1.10 mmol, 1 equiv.) was separately dissolved in THF. Two solutions were refluxed together for 1.5 hours. The solvent was removed. The solid was dissolved in DCM and diethyl ether was added to precipitate out the products. The complex was obtained as a brown solid (47%).[7]

HRMS (ESI) m/z: $[M - Cl]^+$ Calcd for $C_{31}H_{39}ClN_3Ni$ 546.2186; Found 546.2195

Synthesis of Ni-TPA, 2

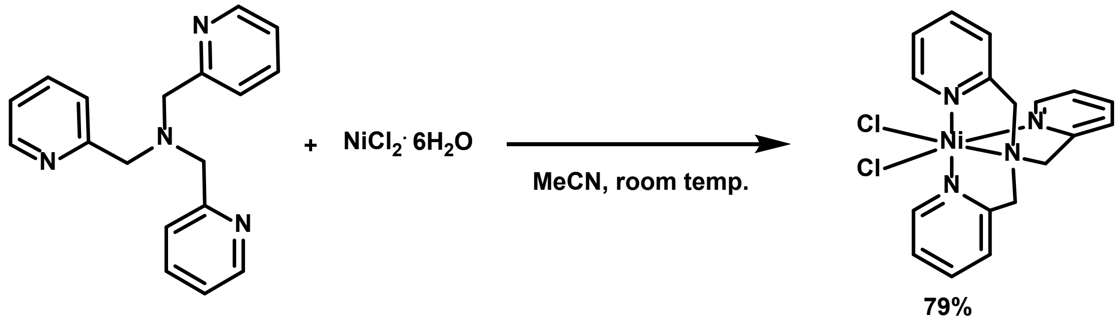

Tris(2-pyridylmethyl)amine (0.2832 g, 1 mmol, 1 equiv.) was dissolved in MeCN and $NiCl_2.6H_2O$ (0.2382 g, 1 mmol, 1 equiv.) was added. The reaction was stirred at room temperature for 3 hours. The solvent was removed under pressure. Then, a few drops of MeCN was added to make a concentrated solution. Et_2O was layered onto the solution and leave for a few days to obtain blue solid. The product was obtained with 0.2523 g (79%).[8]

HRMS (ESI) m/z: $[M - Cl]^+$ Calcd for $C_{18}H_{18}ClN_4Ni$ 383.0568 ; Found 383.0572

Synthesis of Fe-SalenCl$_4$, 3

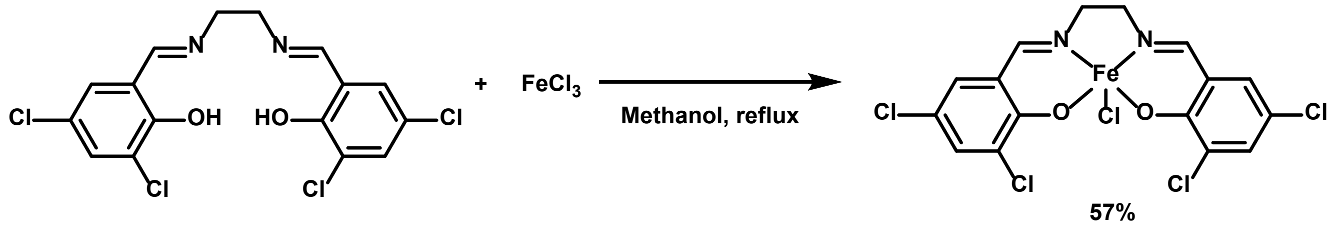

Salen (1.1287 g, 2.78 mmol, 1 equiv) and $FeCl_3$ (0.4812 g, 3 mmol, 1 equiv.) were separately dissolved in methanol (10 mL). Then, two solutions were mixed and refluxed for 5 hours. The solvent was removed under pressure. The solid product was washed by cold Et_2O giving dark brown powder (1.2785 g, 57%).[9]

HRMS (ESI) m/z: $[M - Cl]^+$ Calcd for $C_{16}H_{10}Cl_4FeN_2O_2$ 459.8840; Found 459.8816

2. Characterizations

NMR Spectroscopy

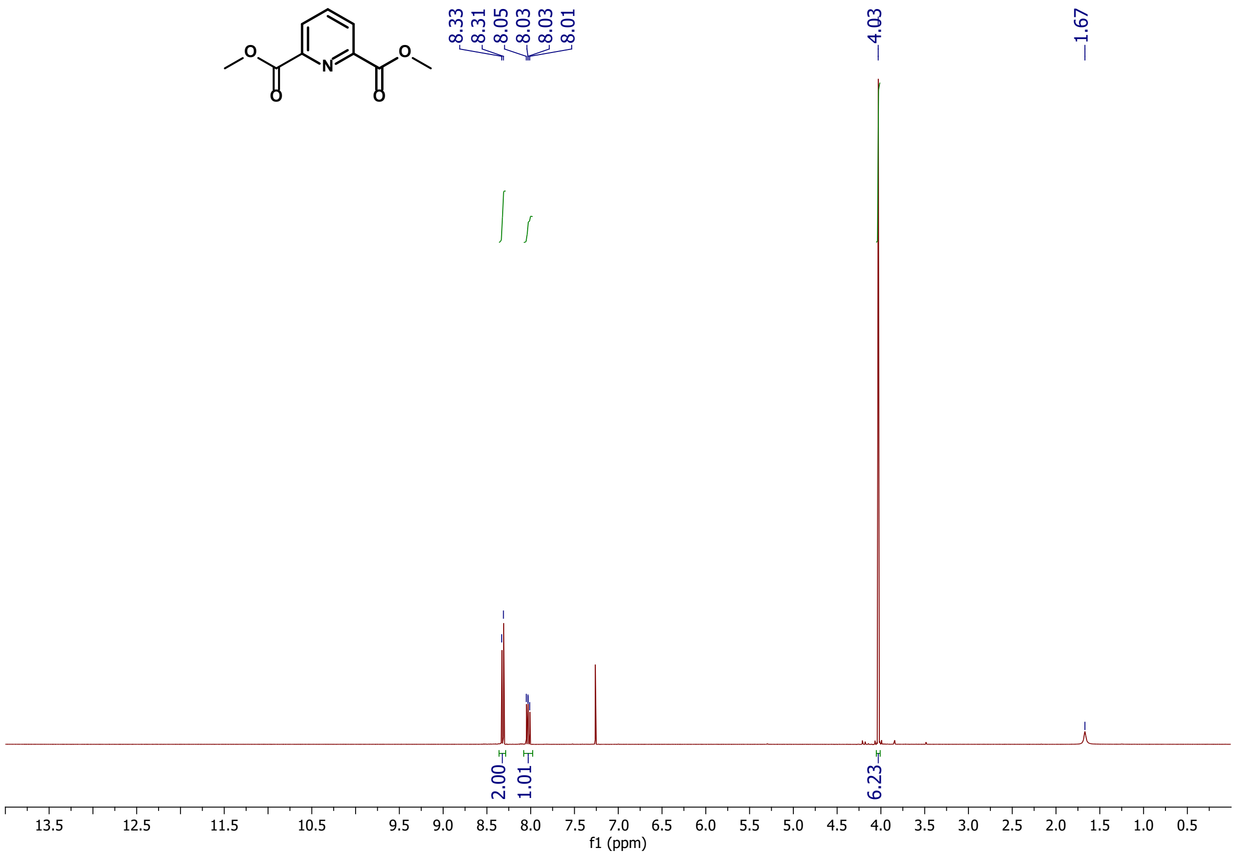

Figure S1 ^{1}H NMR (400 MHz, $CDCl_3$) spectrum of dimethyl 2,6-pyridine dicarboxylate in $CDCl_3$.

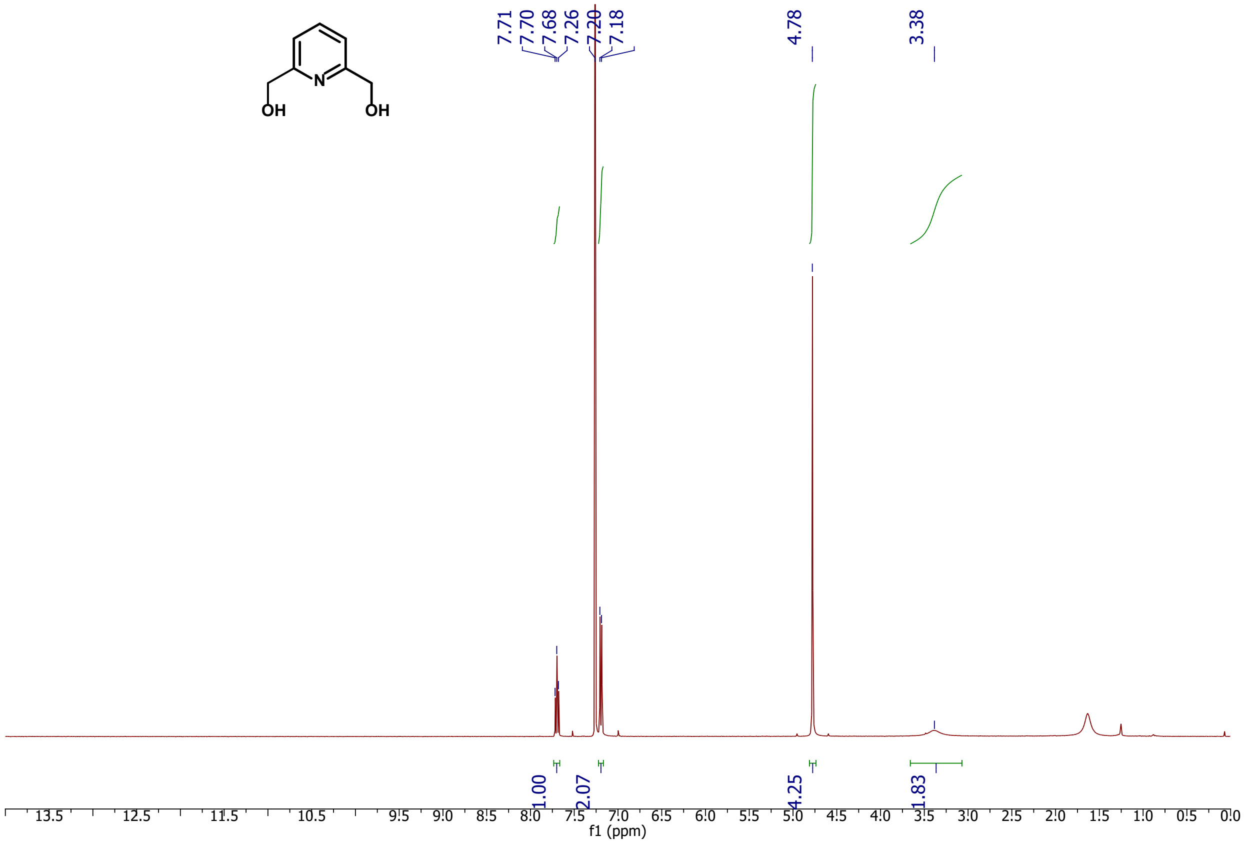

Figure S2 ^{1}H NMR (400 MHz, $CDCl_3$) spectrum of 2,6-pyridine dimethanol in $CDCl_3$.

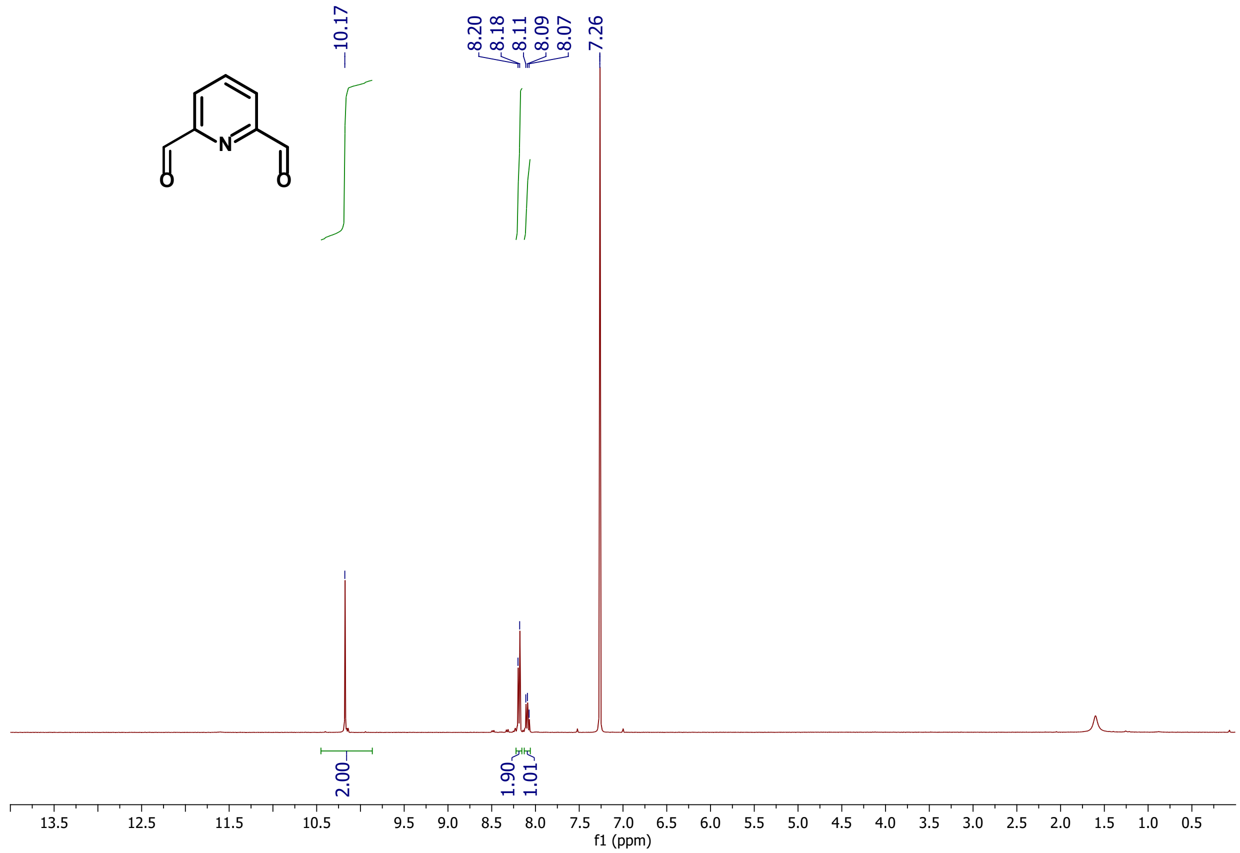

Figure S3 ^{1}H NMR (400 MHz, $CDCl_3$) spectrum of 2,6-pyridine dicarboxaldehyde in $CDCl_3$.

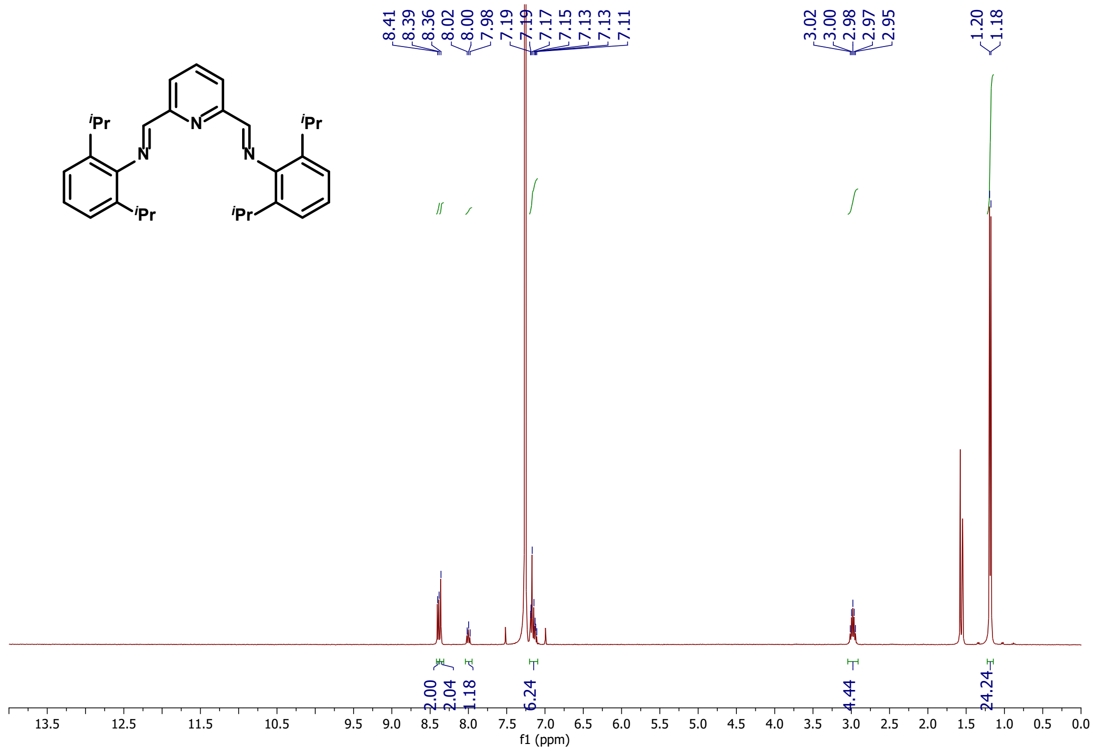

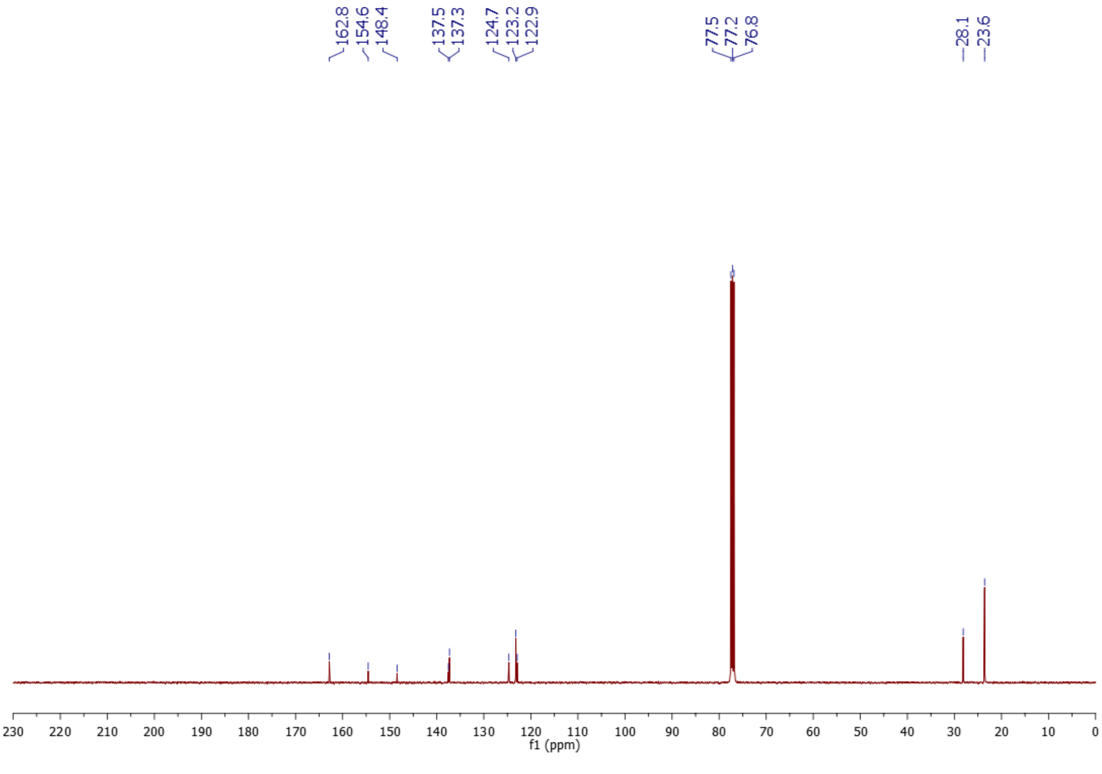

Figure S4 ^{1}H NMR (400 MHz, $CDCl_3$) spectrum (top) and $^{13}C\{^{1}H\}$ NMR (101 MHz, $CDCl_3$) spectrum (bottom) of 2,6-bis(2,6-diisopropylphenyl imino)methyl pyridine

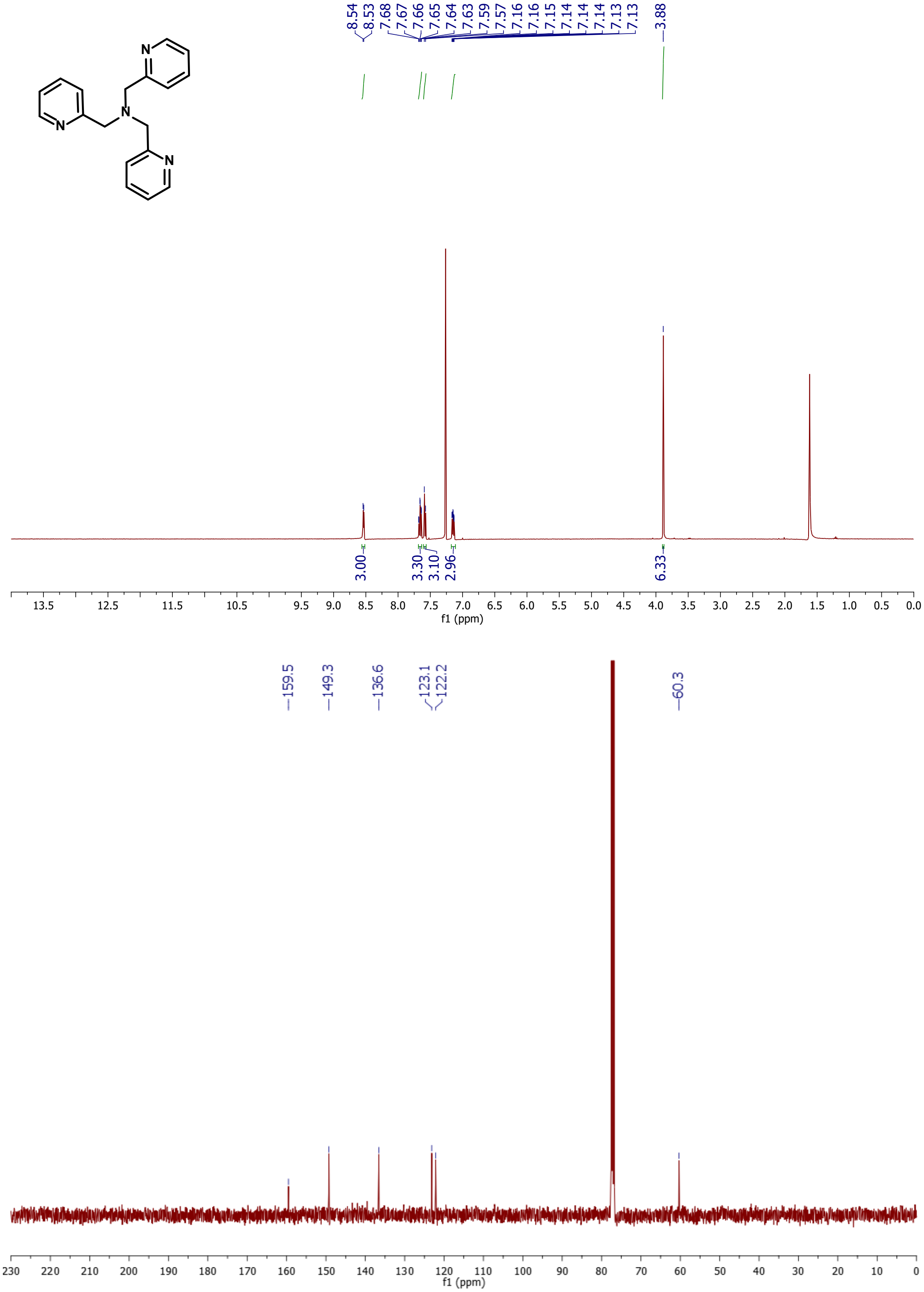

Figure S5 ^{1}H NMR (400 MHz, $CDCl_3$) spectrum (top) and $^{13}C\{^{1}H\}$ NMR (101 MHz, $CDCl_3$) spectrum (bottom) of Tris(2-pyridylmethyl)amine

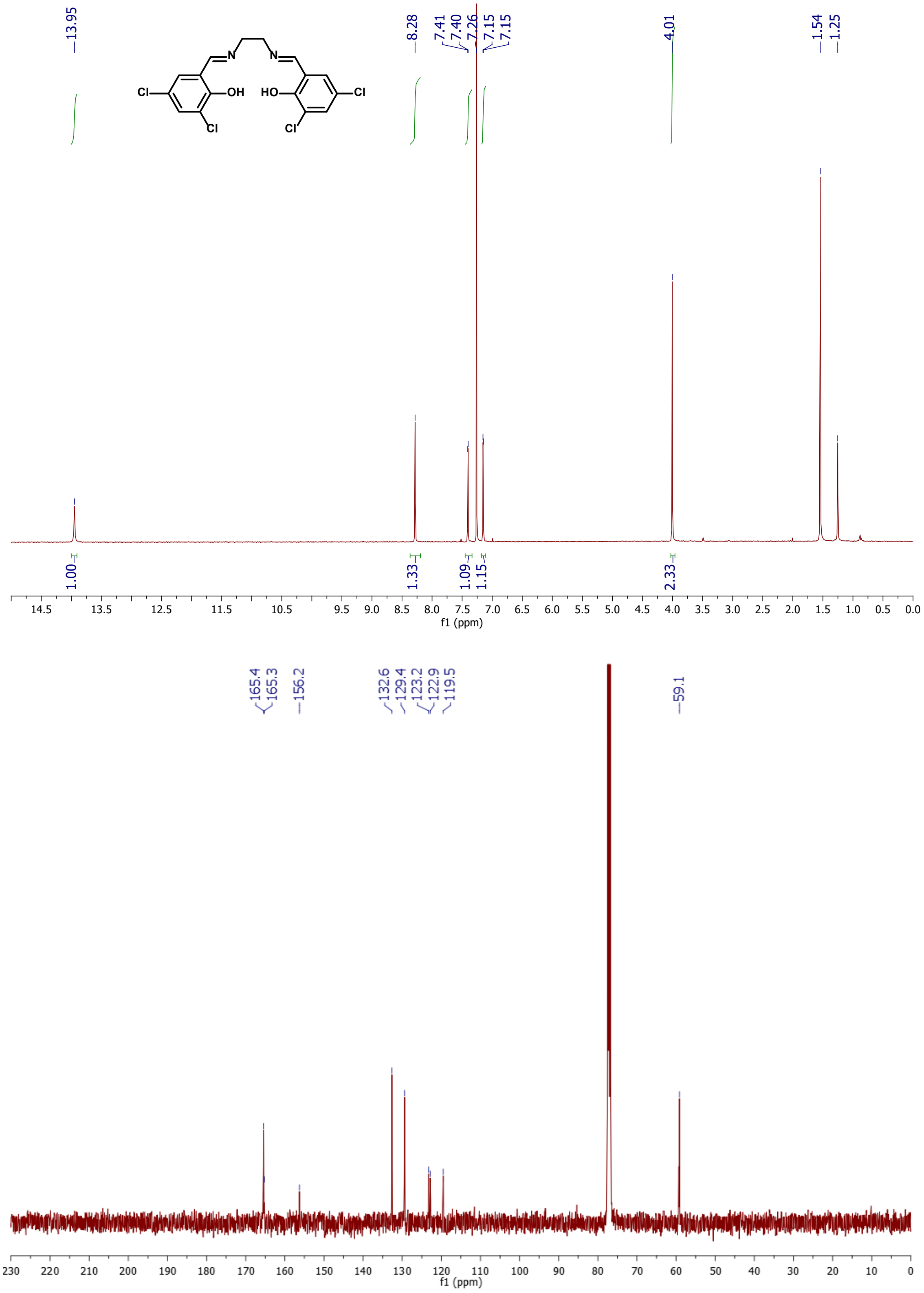

Figure S6 ^{1}H NMR (400 MHz, $CDCl_3$) spectrum (top) and ^{13}C{^{1}H} NMR (101 MHz, $CDCl_3$) spectrum (bottom) of N,N'-bis(3,5-dichlorosalicylidene)-1,2-ethylenediamine

Mass Spectrometry

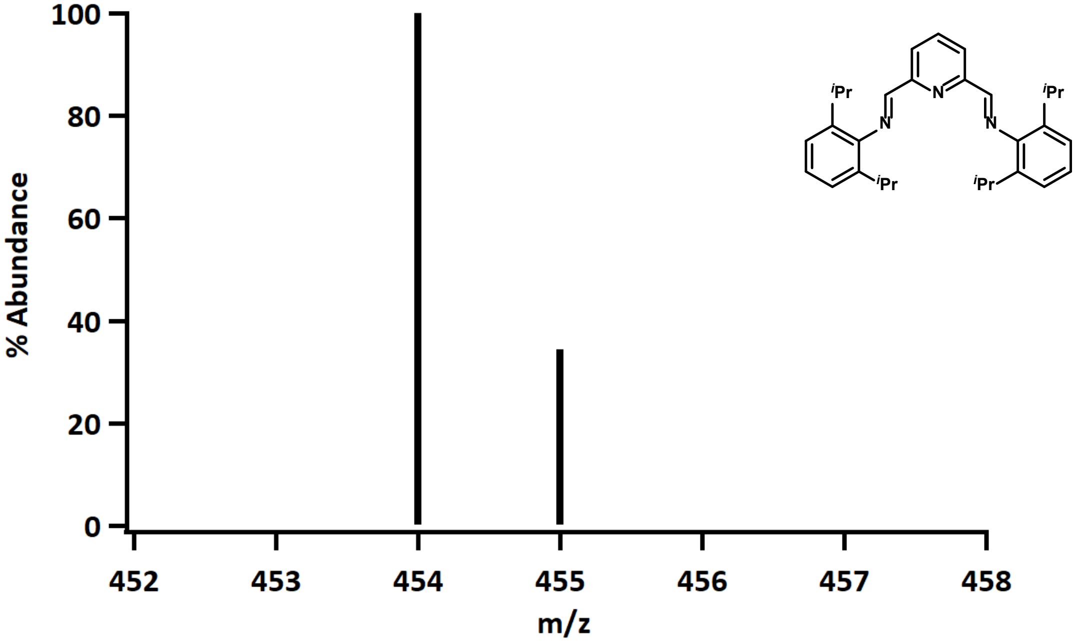

Figure S7 HRMS of 2,6-bis(2,6-diisopropylphenyl imino)methyl pyridine

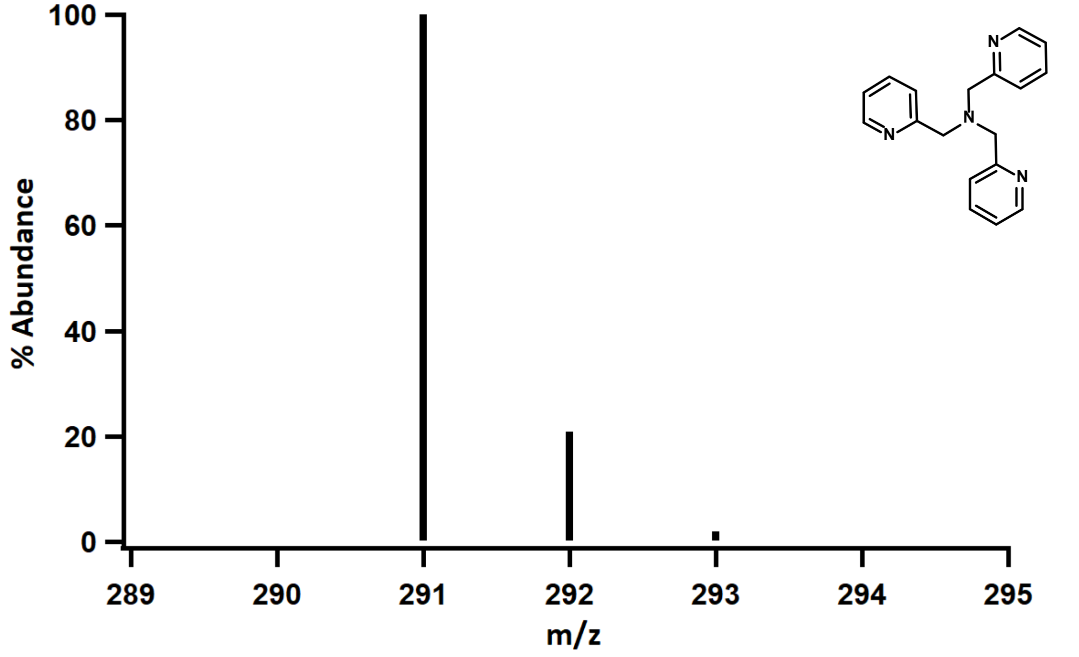

Figure S8 HRMS of Tris(2-pyridylmethyl)amine

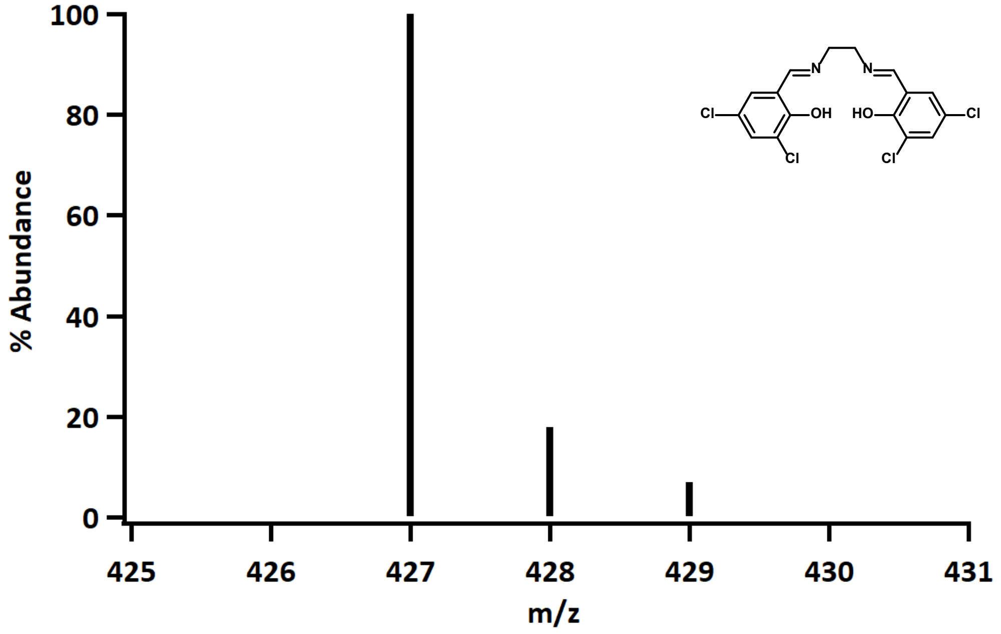

Figure S9 HRMS of N,N'-bis(3,5-dichlorosalicylidene)-1,2-ethylenediamine

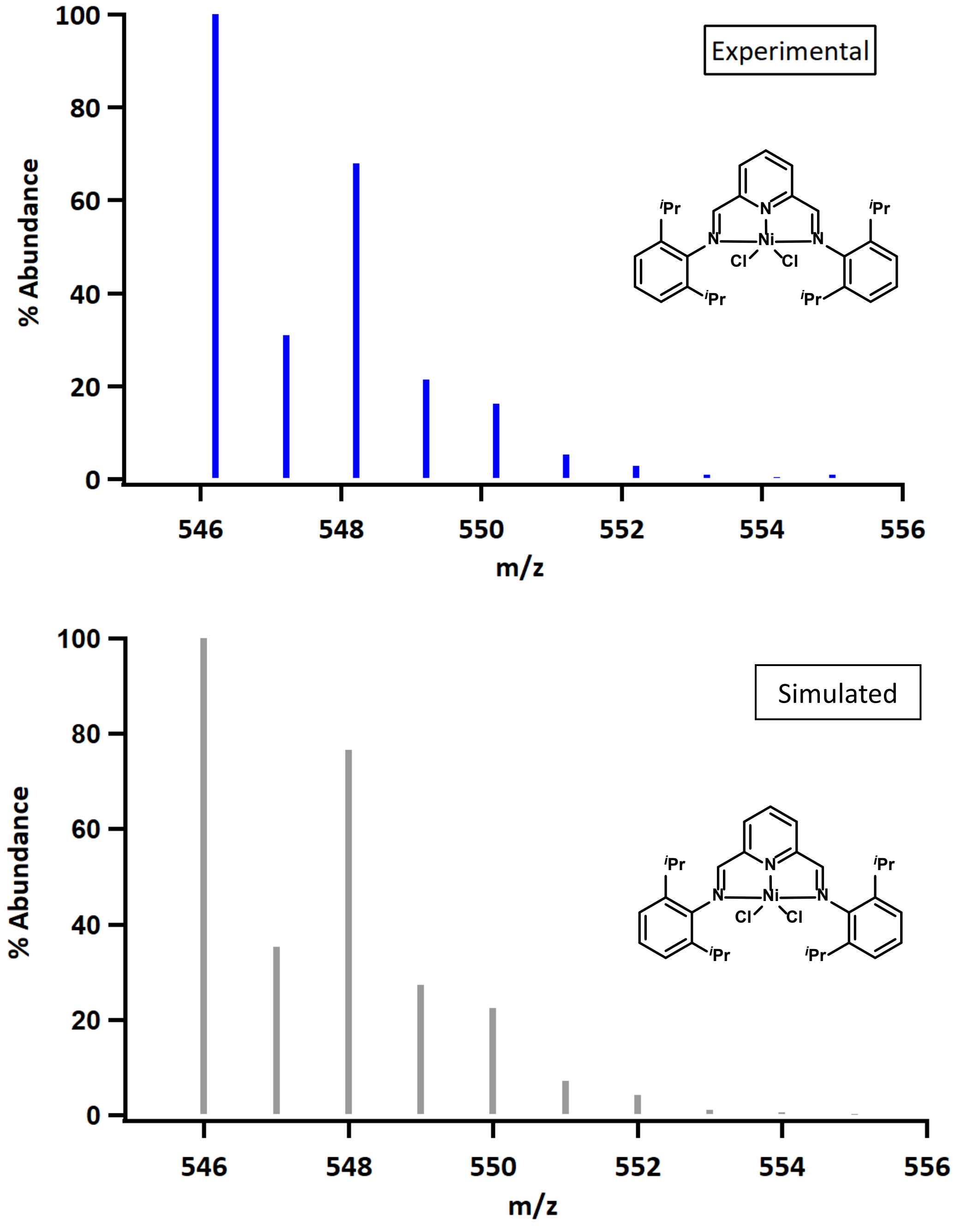

Figure S10 (top) Experimental and (bottom) stimulated HRMS of **1**

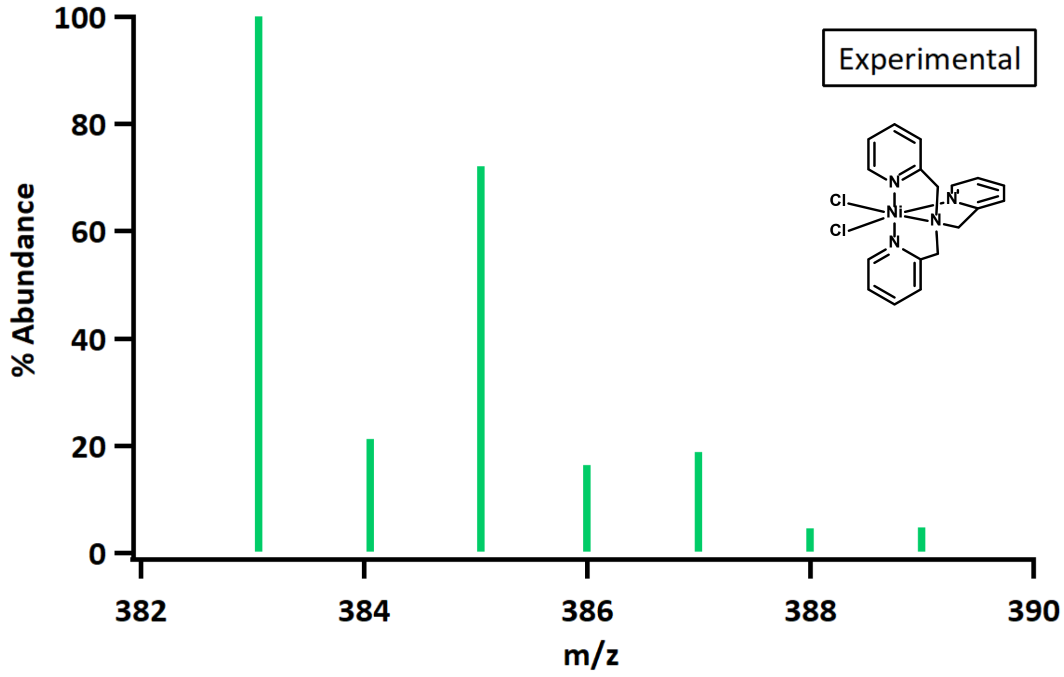

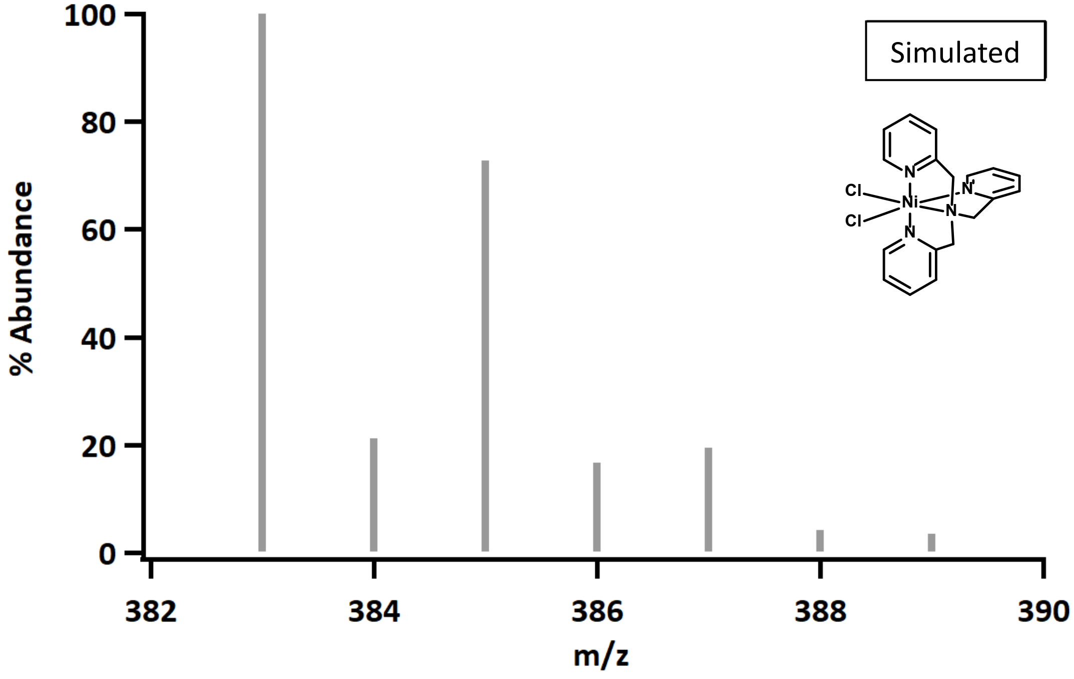

Figure S11 (top) Experimental and (bottom) stimulated HRMS of **2**

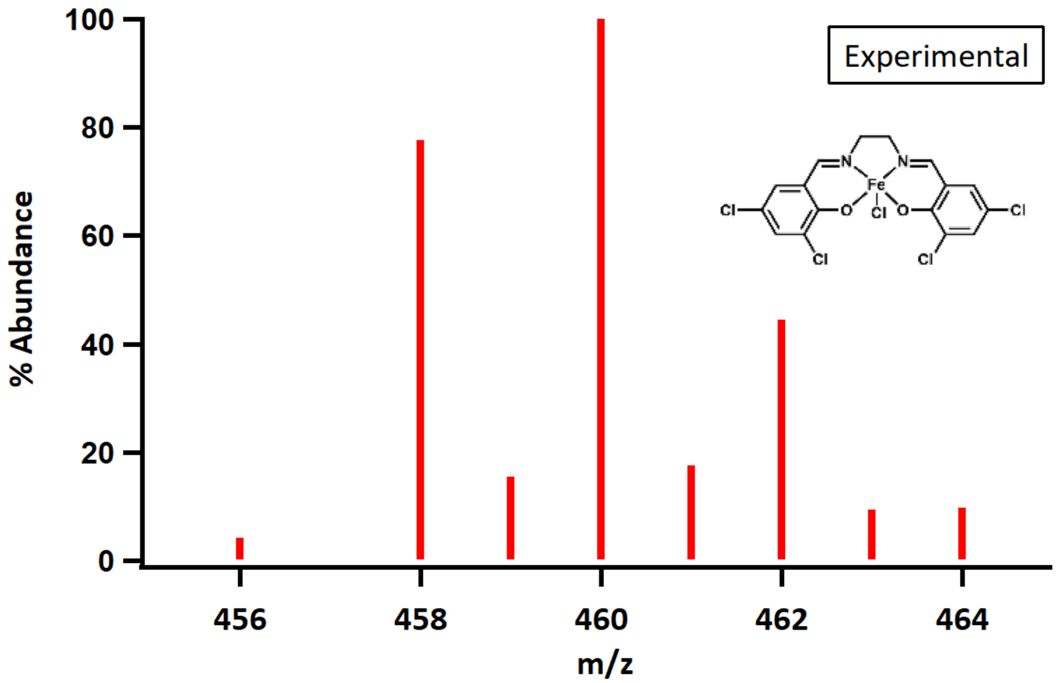

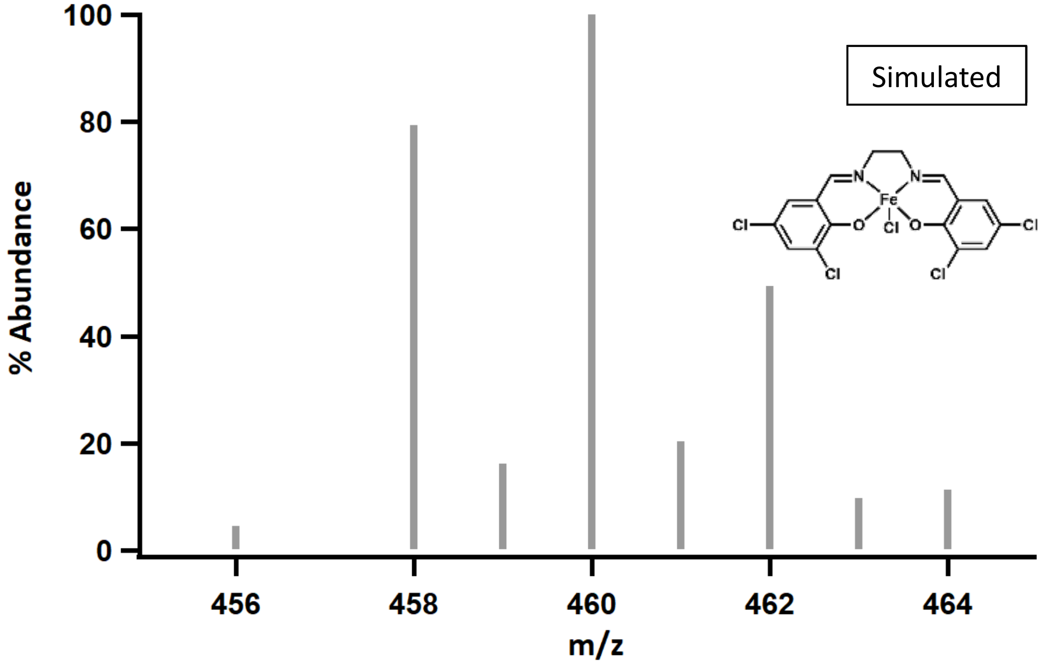

Figure S12 (top) Experimental and (bottom) stimulated HRMS of **3**

UV-vis and IR Spectroscopy

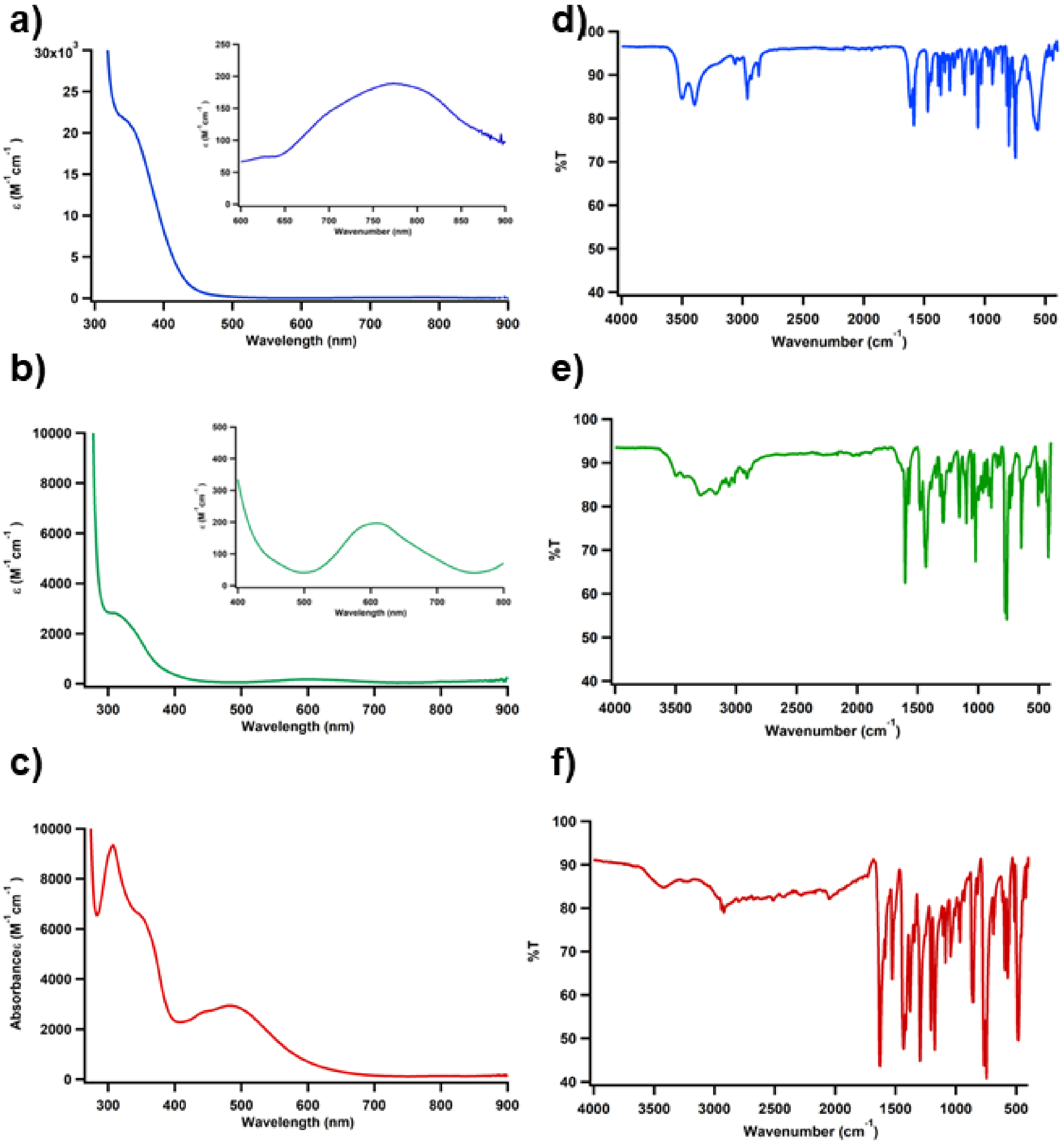

Figure S13 UV-visible spectra of **1** (a), **2** (b), and **3** (c) in MeCN and IR spectra (KBr) of **1** (d), **2** (e), and **3** (f).

3. **Electrochemistry Experimental Procedure**
Cyclic voltammetry

Cyclic voltammetry experiment was carried out in MeCN, unless specified otherwise, with 0.1 M $TBAPF_6$ as electrolyte using glassy carbon as working electrode, platinum wire as counter electrode and Ag/Ag^+ electrode as a pseudo reference electrode. Initial potential was determined by open-circuit potential measurement and used as the scan starting point. All the scans were performed in the reductive direction of the starting potential. Ferrocene was added to the end of each experiment to set $Fe(C_5H_5)_2^{+/0}$ couple as a reference. All experiments were performed under Ar and CO_2 atmosphere. Scan rates were set to range from 25 to 10,000 mV/s.

Electrochemical Carboxylation Reactions

i) General Procedure of Electrochemical Carboxylation Reaction of Benzyl Chloride

Chemicals were added to an undivided cell in the following order; 0.5 mmol of $TBAPF_6$, 0.25 mmol of benzyl chloride, 2 mol% of Ni-PDI(^{i}Pr), 2 mol% of DMAP, 0.325 mmol of $MgBr_2$. NMP was then added to the reaction mixture (8 mL). CO_2 was purged into the solvent of the reaction for 15 minutes. The constant electric current (5 mA) was applied to the carbon cloth electrodes (1 cm x 2 cm). The reaction was stirred for 4 hours under applied current with CO_2 flow of 1 atm in the headspace. To work up, 2 M HCl (20 mL) was added to the solution and extracted by Et_2O (20 mL x 3), followed by brine (20 mL). Solvent was reduced under pressure to yield light yellow oil.

ii) Quantitative characterization of electrochemical carboxylation products

After working up the reaction, all of the reaction mixture, 100 μL of 50 mM 1,3,5-trimethoxybenzene in MeCN (HPLC grade) were mixed in 2 mL volumetric flask and MeCN were added to adjust the volume to 2 mL. The solution was then filtered through syringe filter into 2 mL GC-Vial.

iii) Faradaic Efficiency calculation

Faradaic efficiency is used to describe the overall selectivity of a product from electrochemical reactions. It is evaluated from the mole of product obtained relative to the expected product from the total charge passes consumed in the reactions.[9,10] Turnover number of catalysts indicated the maximum use which can be made by the catalyst in certain chemical reactions.[11]

$$\mathrm{FE}(\%) = \frac{Q_{\mathrm{prod}}}{Q_{\mathrm{total}}} \times 100 \qquad \mathrm{FE}_{\mathrm{liquid}}(\%) = \frac{N \times n \times F}{Q_{\mathrm{total}}} \times 100$$

Equation 1. Equations for faradaic efficiency calculation, Q_{prod} is the charge consumed for generation of a product and Q_{total} is the total charge passed during electrolysis. N is the number of moles of liquid product formed, n is the number of moles of e^- required for 1 mol of product formation, and F is the Faraday constant (~96485 C/mol).[10]

Faradaic Effiency was calculated based on the applied constant current (5mA), which was applied to the reaction for 4 hours (3600×4 seconds). Q_{total} was calculated as 72 C (5×10^{-3} A × 4 × 3600 s). Q_{prod} was calculated for each product based on the yield obtained from GC analysis, using n = 2.

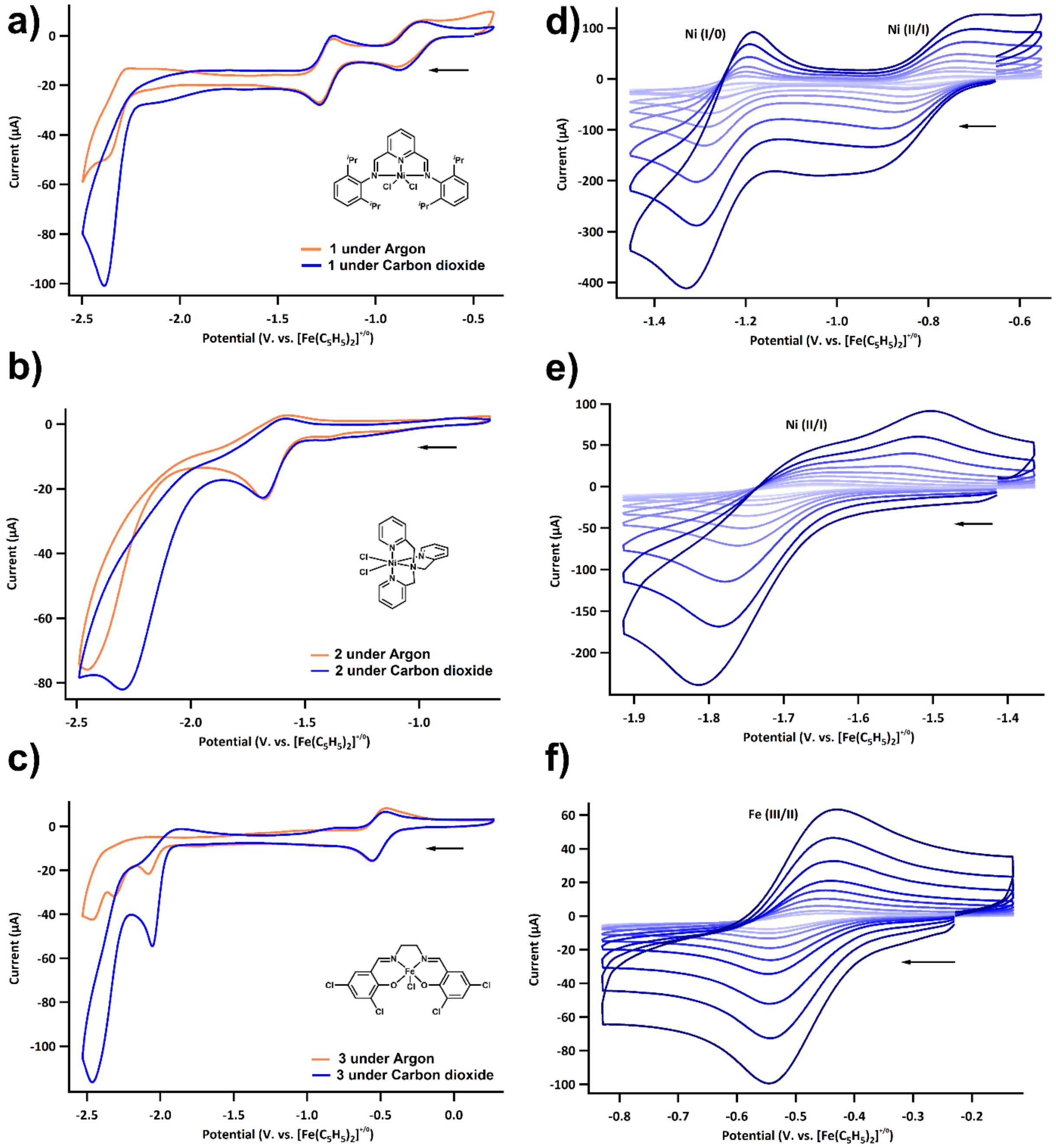

Figure S14 a) cyclic voltammogram of **1** under Ar and carbon dioxide, d) Scan rate-dependent cyclic voltammogram of **1** under carbon dioxide with scan rate of 25 mV/s to 10,000 mV/s, b) cyclic voltammogram of **2** under Ar and carbon dioxide, e) Scan rate-dependent cyclic voltammogram of **2** under carbon dioxide with scan rate of 25 mV/s to 10,000 mV/s, c) cyclic voltammogram of **3** under Ar and carbon dioxide, f) Scan rate-dependent cyclic voltammogram of

3 under carbon dioxide with scan rate of 25 mV/s to 10,000 mV/s. Working electrode, counter electrode, and reference electrode are glassy carbon, Pt wire, and Ag/Ag+ pseudo reference electrode, respectively. Cyclic voltammograms were recorded in MeCN with 0.1 M $TBAPF_6$ and 1 mM of **1**, **2**, or **3**, at the scan rate of 100 mV/s, unless otherwise noted.

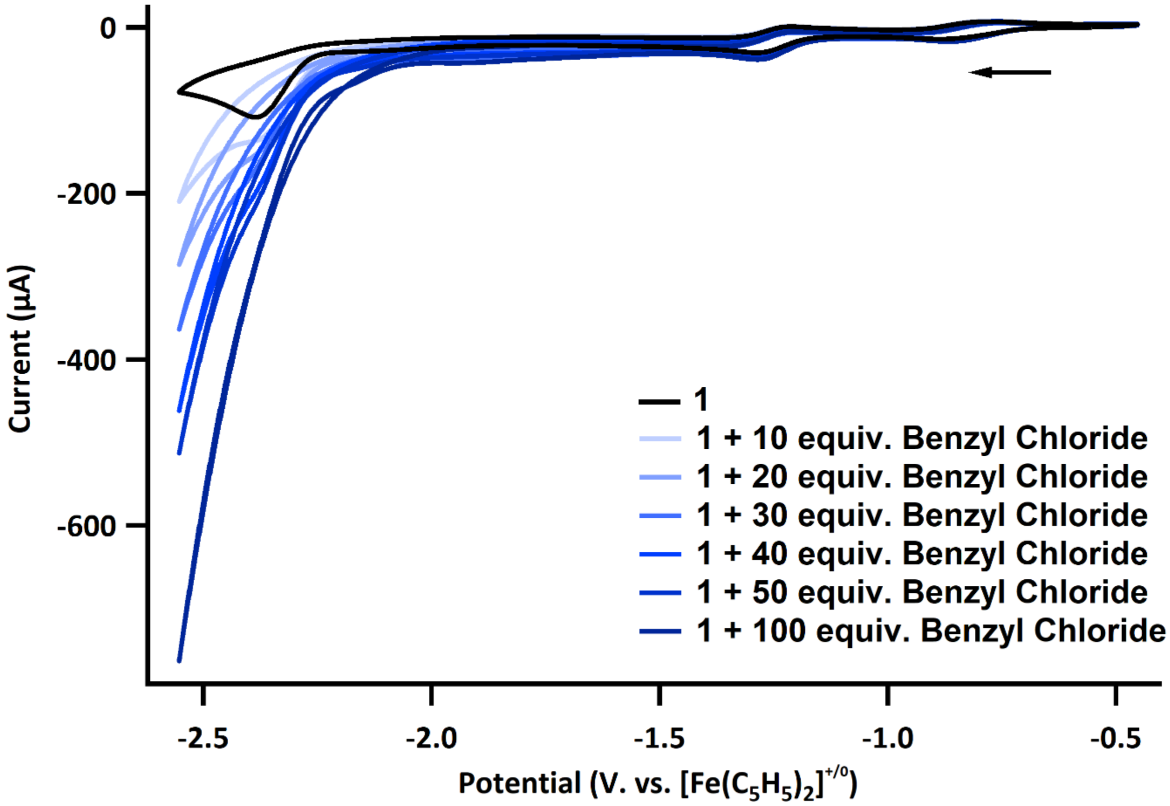

Figure S15 Cyclic voltammogram of **1** under CO_2 (black), with additional benzyl chloride (blue lines). Working electrode, counter electrode, and reference electrode are glassy carbon, Pt wire, and Ag/Ag+ pseudo reference electrode, respectively. Cyclic voltammograms were recorded in MeCN with 0.1 M $TBAPF_6$ and 1 mM of **1**, **2**, or **3**, at the scan rate of 100 mV/s.

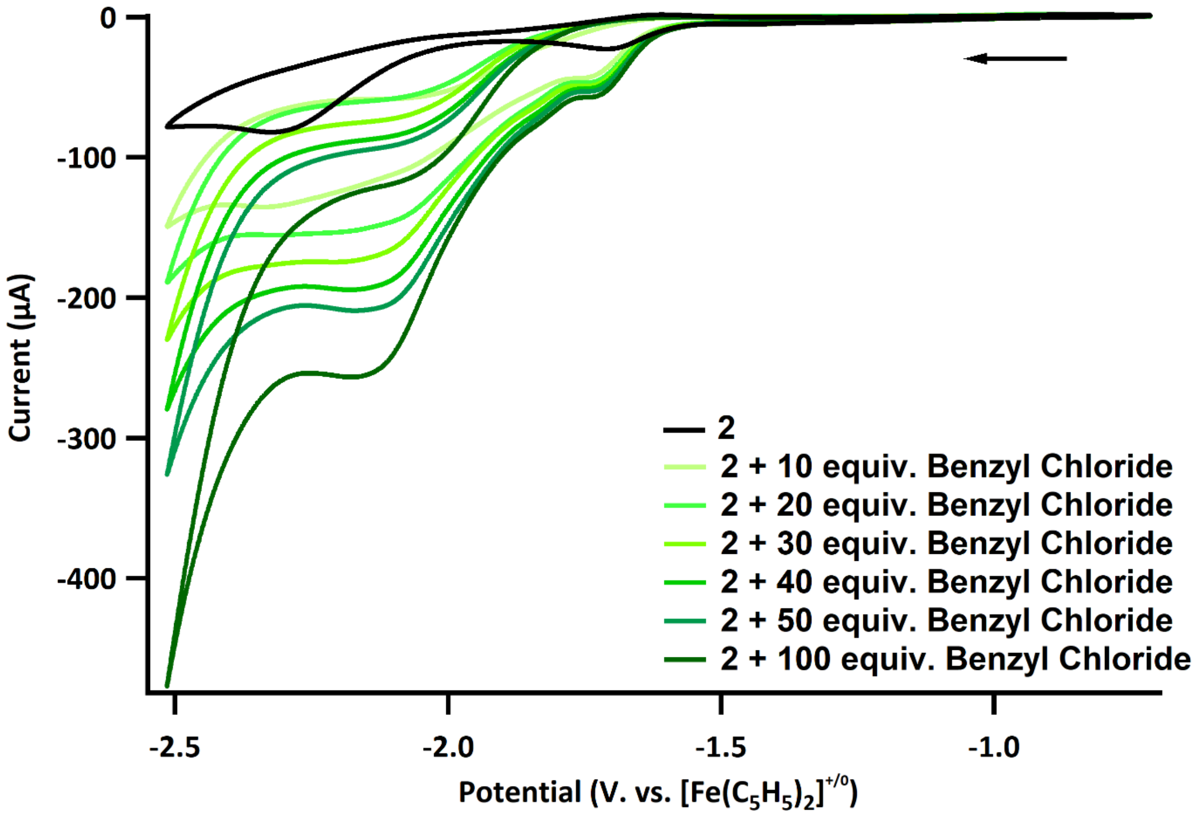

Figure S16 Cyclic voltammogram of **2** under CO_2 (black), with additional benzyl chloride (green lines). Working electrode, counter electrode, and reference electrode are glassy carbon, Pt wire, and Ag/Ag^+ pseudo reference electrode, respectively. Cyclic voltammograms were recorded in MeCN with 0.1 M $TBAPF_6$ and 1 mM of **1**, **2**, or **3**, at the scan rate of 100 mV/s.

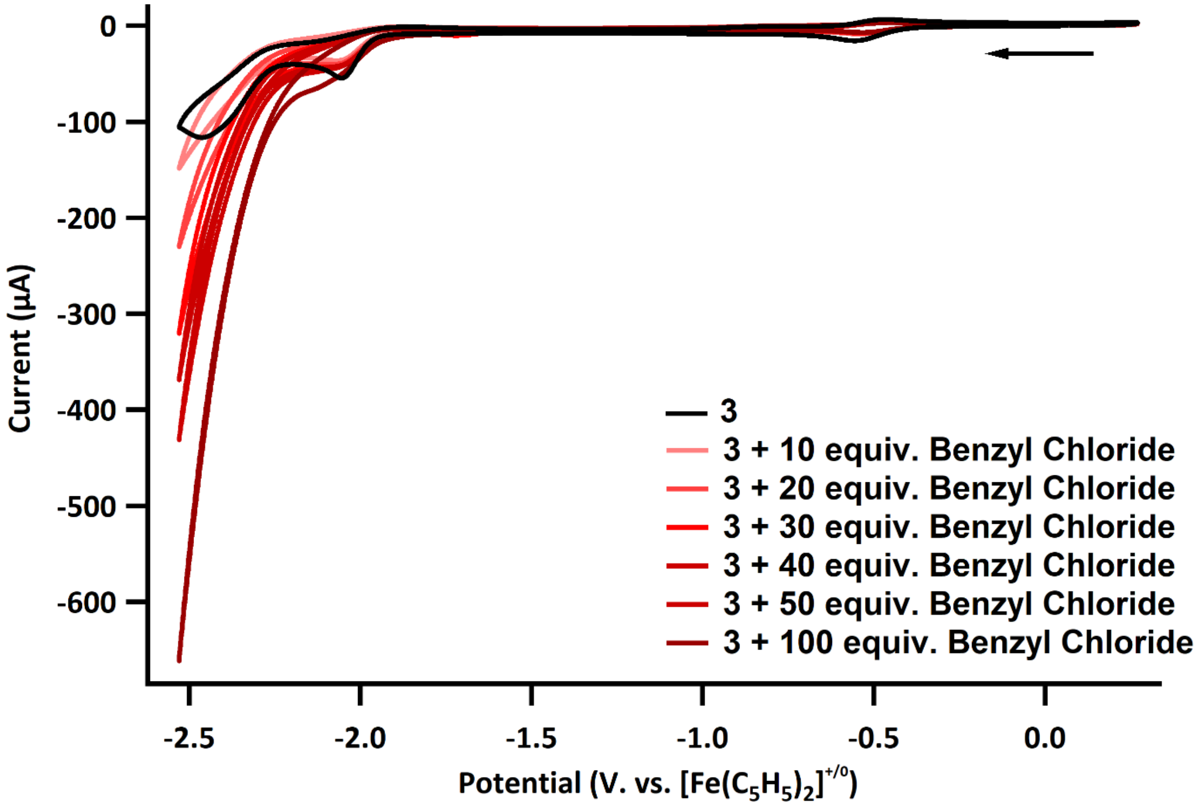

Figure S17 Cyclic voltammogram of **3** under CO_2 (black), with additional benzyl chloride (red lines). Working electrode, counter electrode, and reference electrode are glassy carbon, Pt wire, and Ag/Ag^+ pseudo reference electrode, respectively. Cyclic voltammograms were recorded in MeCN with 0.1 M $TBAPF_6$ and 1 mM of **1**, **2**, or **3**, at the scan rate of 100 mV/s.

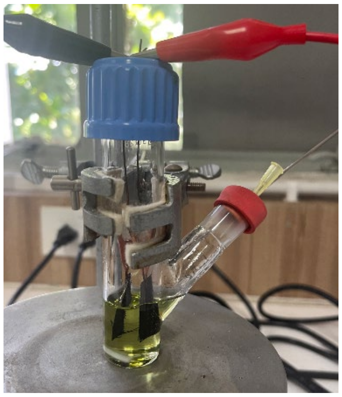

Figure S18 Electrolysis set up with one-compartment cell, two carbon electrodes, and a flow of 1 atm as a blanket on top of the reaction mixture

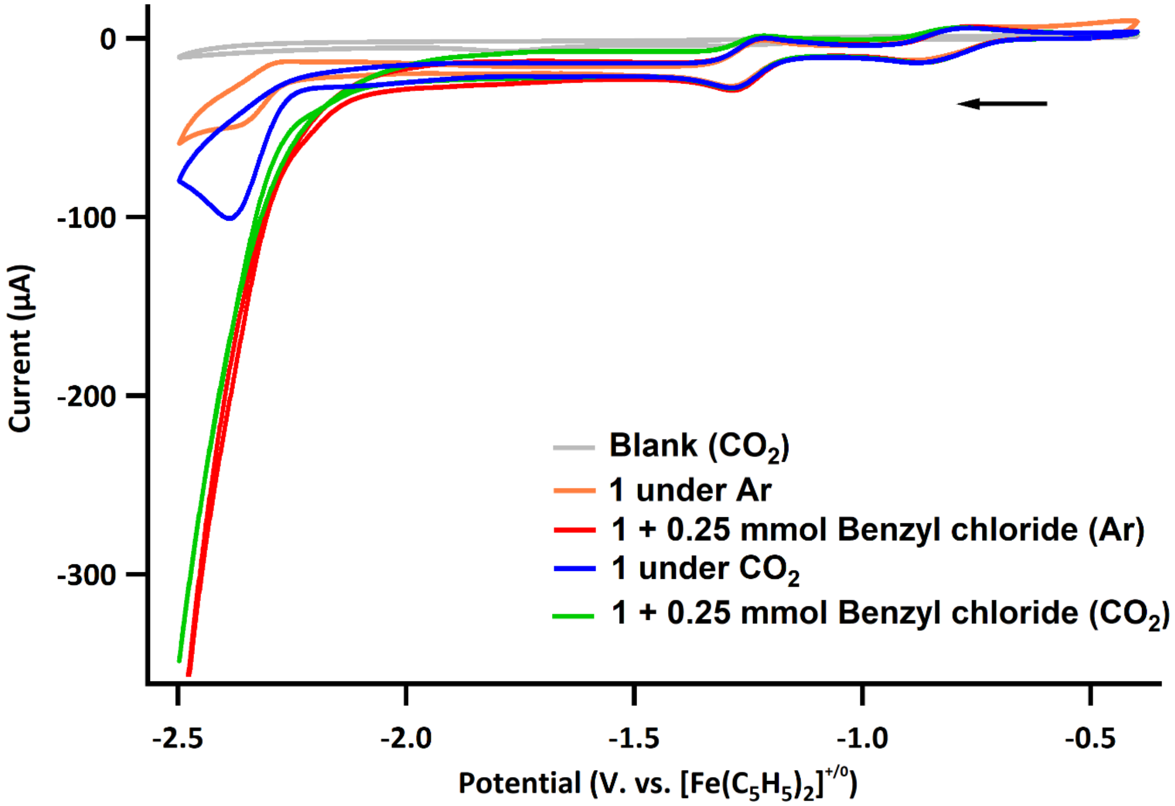

Figure S19 Cyclic voltammograms of **1** under Ar and CO_2, and **1** with 0.25 mmol benzyl chloride under Ar and CO_2. Working electrode, counter electrode, and reference electrode are glassy carbon, Pt wire, and Ag/Ag^+ pseudo reference electrode, respectively. Cyclic voltammograms were recorded in MeCN with 0.1 M $TBAPF_6$ and 1 mM of 1, at the scan rate of 100 mV/s.

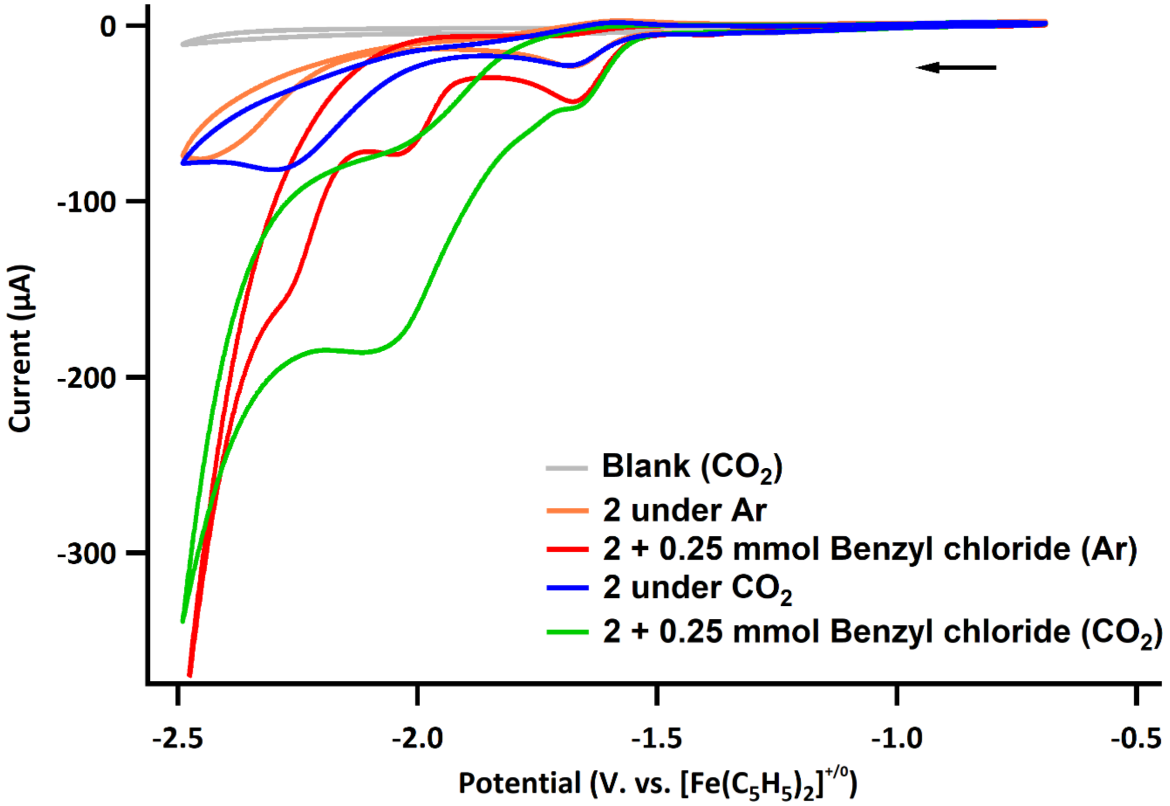

Figure S20 Cyclic voltammograms of **2** under Ar and CO_2, and **2** with 0.25 mmol benzyl chloride under Ar and CO_2. Working electrode, counter electrode, and reference electrode are glassy carbon, Pt wire, and Ag/Ag^+ pseudo reference electrode, respectively. Cyclic voltammograms were recorded in MeCN with 0.1 M $TBAPF_6$ and 1 mM of **2**, at the scan rate of 100 mV/s.

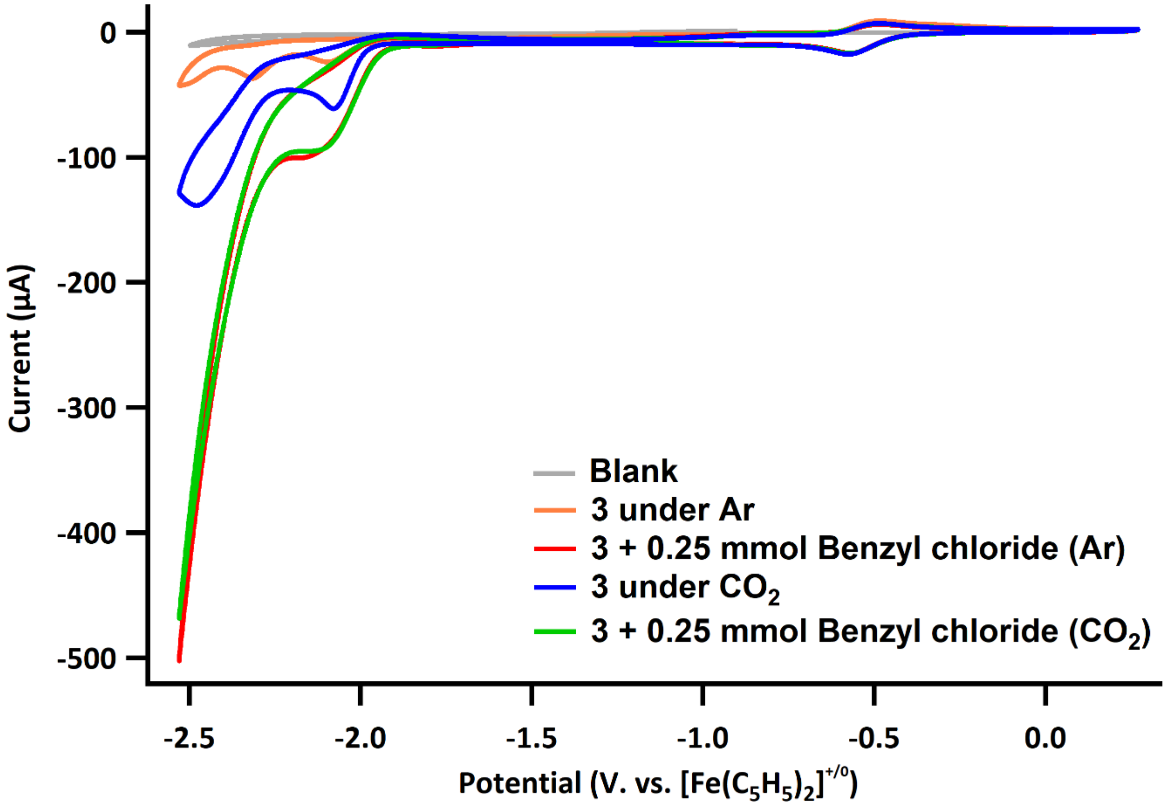

Figure S21 Cyclic voltammograms of **3** under Ar and CO_2, and **3** with 0.25 mmol benzyl chloride under Ar and CO_2. Working electrode, counter electrode, and reference electrode are glassy carbon, Pt wire, and Ag/Ag^+ pseudo reference electrode, respectively. Cyclic voltammograms were recorded in MeCN with 0.1 M $TBAPF_6$ and 1 mM of **3**, at the scan rate of 100 mV/s.

4. **References**

1. Chen, A. Y.; Thomas, P. W.; Stewart, A. C.; Bergstrom, A.; Cheng, Z.; Miller, C.; Bethel, C. R.; Marshall, S. H.; Credille, C. V.; Riley, C. L.; Page, R. C.; Bonomo, R. A.; Crowder, M. W.; Tierney, D. L.; Fast, W.; Cohen, S. M., Dipicolinic Acid Derivatives as Inhibitors of New Delhi Metallo-β-lactamase-1. *J. Med. Chem.* **2017,** *60* (17), 7267-7283.
2. Clarke, C. J.; Bui-Le, L.; Hallett, J. P.; Licence, P., Thermally-Stable Imidazolium Dicationic Ionic Liquids with Pyridine Functional Groups. *ACS Sustainable Chemistry & Engineering* **2020,** *8* (23), 8762-8772.

   Bayeh, Y.; Osuský, P.; Yutronkie, N. J.; Gyepes, R.; Sergawie, A.; Hrobárik, P.; Clérac, R.; Thomas, M., Spin state of two mononuclear iron(II) complexes of a tridentate bis(imino)pyridine N-donor ligand: Experimental and theoretical investigations. *Polyhedron* **2022,** *227*, 116136.
3. Britovsek, G. J. P.; Bruce, M.; Gibson, V. C.; Kimberley, B. S.; Maddox, P. J.; Mastroianni, S.; McTavish, S. J.; Redshaw, C.; Solan, G. A.; Strömberg, S.; White, A. J. P.; Williams, D. J., Iron and Cobalt Ethylene Polymerization Catalysts Bearing 2,6-Bis(Imino)Pyridyl Ligands: Synthesis, Structures, and Polymerization Studies. *J. Am. Chem. Soc.* **1999,** *121* (38), 8728-8740.
4. Beni, A.; Dei, A.; Laschi, S.; Rizzitano, M.; Sorace, L., Tuning the Charge Distribution and Photoswitchable Properties of Cobalt–Dioxolene Complexes by Using Molecular Techniques. *Chem. Eur. J.* **2008,** *14* (6), 1804-1813.
5. Li, H.; Xi, D.; Niu, Y.; Wang, C.; Xu, F.; Liang, L.; Xu, P., Design, synthesis and biological evaluation of cobalt(II)-Schiff base complexes as ATP-noncompetitive MEK1 inhibitors. *J. Inorg. Biochem.* **2019,** *195*, 174-181.
6. Reed, B. R.; Stoian, S. A.; Lord, R. L.; Groysman, S., The aldimine effect in bis(imino)pyridine complexes: non-planar nickel(i) complexes of a bis(aldimino)pyridine ligand. *ChemComm.* **2015,** *51* (30), 6496-6499.
7. Rebolledo-Chávez, J. P. F.; Cruz-Ramírez, M.; Ramírez-Palma, D. I.; Ocampo-Hernández, J.; Mendoza, A.; Cortés-Guzmán, F.; Ortiz-Frade, L., Electrochemical mechanism of CO_2 reduction mediated by Ni^{II}(tpa) (tpa = tris(2-pyridylmethyl)amine) complexes: An integral view. *Electrochim. Acta* **2021,** *400*, 139465.

8. Cozzolino, M.; Leo, V.; Tedesco, C.; Mazzeo, M.; Lamberti, M., Salen, salan and salalen iron(iii) complexes as catalysts for CO_2/epoxide reactions and ROP of cyclic esters. *Dalton Trans.* **2018,** *47* (37), 13229-13238.
9. Kempler, P. A.; Nielander, A. C. Reliable reporting of Faradaic efficiencies for electrocatalysis research. *Nat. Commun.* **2023**, *14* (1), 1158.
10. Dutta, N.; Bagchi, D.; Chawla, G.; Peter, S. C. A Guideline to Determine Faradaic Efficiency in Electrochemical CO_2 Reduction. *ACS Energy Lett.* **2024**, *9* (1), 323-328.
11. Kozuch, S.; Martin, J. M. L. "Turning Over" Definitions in Catalytic Cycles. *ACS Catal.* **2012**, *2* (12), 2787-2794.
